# Persistent Symptoms in SARS‐CoV‐2‐Infected and Non‐Infected Household Members: A Prospective Cohort Study

**DOI:** 10.1002/jmv.70727

**Published:** 2025-12-06

**Authors:** Angelique M. A. M. Winkel, Bastienne A. de Jonghe, Coen R. Lap, Mildred E. Haverkort, Judith G. C. Sluiter‐Post, Sjoerd M. Euser, Dirk Eggink, Suzanne E. Geerlings, Adriana Tami, Menno D. de Jong, Steven F. L. van Lelyveld, Marianne A. van Houten

**Affiliations:** ^1^ Spaarne Gasthuis Academy Hoofddorp The Netherlands; ^2^ Department of Medical Microbiology Amsterdam University Medical Centre Amsterdam The Netherlands; ^3^ Department of Pediatric Immunology and Infectious Diseases Wilhelmina Children's Hospital and University Medical Centre Utrecht Utrecht The Netherlands; ^4^ Department of Infectious Disease Control Public Health Services Kennemerland Haarlem The Netherlands; ^5^ Regional Public Health Laboratory Kennemerland Haarlem The Netherlands; ^6^ Centre for Infectious Disease Control National Institute for Public Health and the Environment Bilthoven The Netherlands; ^7^ Department of Internal Medicine Amsterdam Institute for Infectious Diseases and Immunity, Amsterdam UMC Amsterdam The Netherlands; ^8^ Department of Medical Microbiology and Infection Prevention University Medical Center Groningen University of Groningen Groningen The Netherlands; ^9^ Department of Internal Medicine Spaarne Gasthuis Hospital Haarlem/Hoofddorp The Netherlands; ^10^ Department of Pediatrics Spaarne Gasthuis Haarlem/Hoofddorp The Netherlands

**Keywords:** COVID‐19, household, post‐acute sequelae of COVID‐19 (PASC), long‐COVID, SARS‐CoV‐2

## Abstract

This prospective study assessed the prevalence, type, and consequences of persistent symptoms following a nonhospitalized SARS‐CoV‐2 infection by comparing infected and noninfected children and adults of Dutch households. Two comparable prospective household studies were conducted during two pandemic phases. At baseline, all household members were tested for SARS‐CoV‐2 with 10 consecutive saliva samples during a 6‐week period using RT‐PCR. Questionnaires assessing persistent symptoms, health‐related quality of life (HRQoL), anxiety, and depressive symptoms were collected at 6 and 12 months. Of the 297 included participants (median age 34 years, IQR 12–48), 201 (67.7%) tested positive for SARS‐CoV‐2. At 6 months, only one child reported persistent symptoms. SARS‐CoV‐2‐infected adults (> 18 years) reported more pulmonary symptoms (15.2% vs. 3.4%, *p* = 0.023), and tended to report more fatigue (12.8% vs. 3.4%, *p *= 0.061) and exertion‐related symptoms (8.8% vs. 1.7%, *p *= 0.107) compared to the negative adults. Adult participants with persistent symptoms reported decreased HRQoL and increased anxiety and depressive symptoms. This study found that SARS‐CoV‐2‐positive adults tended to have higher prevalence of respiratory symptoms, fatigue, and exertion‐related symptoms 6 months after SARS‐CoV‐2 infection, whereas children rarely reported persistent symptoms. Persistent symptoms were associated with a reduced HRQoL and increased anxiety and depression.

**Abbreviations**
COVID‐19: coronavirus disease 2019
HADS: hospital anxiety and depression scale
HRQoL: health‐related quality of life
MCS: mental component score
PCS: physical component score
SARSLIVA: SARS‐CoV‐2 in saLIVA
SARS‐CoV‐2: severe acute respiratory syndrome coronavirus 2
SF‐36: 36‐Item Short Form Health Survey
UMC: University Medical Center
VOC: variant of concern
WHO: World Health Association

## 1 Introduction

Persistent symptoms are frequently reported after a SARS‐CoV‐2 infection. Estimates range from 7.5% to 35% in nonhospitalized and 53% to 72.5% in hospitalized adults [1, 2, 3]. In nonhospitalized children, estimates range from 0% to 40.0% [4, 5, 6]. An array of persistent symptoms has been reported; however, these symptoms are common in the general population, and many studies lack a control group. This may lead to an overestimation of persistent symptoms attributed to a SARS‐CoV‐2 infection. A recent systematic review reporting on persistent symptoms in children and adults after a SARS‐CoV‐2 infection only included studies with a control group [7]. It showed that the majority of reported symptoms were equally prevalent in SARS‐CoV‐2 patients compared to negative controls [7]. However, as noted in the article, the limited number of studies that could be included in the analysis necessitates a cautious interpretation of these findings, which underscores the need for additional comparative studies. Furthermore, this limited number of available studies included heterogeneous control groups [7]. This heterogeneity introduces variability in lifestyle factors, including differences in approaches to managing and addressing symptoms among cases and controls, which limits comparability. These confounders may undermine the ability to distill the true effect of SARS‐CoV‐2 infection on the prevalence of persistent symptoms. Studies conducted in household settings mitigate these challenges by leveraging shared genetics, environmental exposures, and lifestyle factors, resulting in more homogeneous control groups and enabling clear comparisons.

Therefore, this study analyzed the prevalence of persistent symptoms 6 and 12 months after a SARS‐CoV‐2 infection in nonhospitalized children and adults of Dutch households compared to uninfected household members. Moreover, this study aims to evaluate health‐related quality of life (HRQoL), anxiety, and depressive symptoms in participants with and without persistent symptoms, and to evaluate possible associations between patient characteristics and persistent symptoms.

## 2 Methods

This prospective questionnaire study is a 12‐month follow‐up of the SARSLIVA 1.0 (SL1) and SARSLIVA 2.0 cohort (SL2). These studies, with similar study designs, investigated SARS‐CoV‐2 household transmission during different stages of the COVID‐19 pandemic [8, 9]. These cohorts consist of participants aged 0–65 years from Dutch households. Participants from the SL1 cohort were included between October and December 2020, when the wild‐type variant was dominant in an immune naïve population. Participants from the SL2 cohort were included between March and April 2022, during the dominance of the Omicron BA.2 variant of concern (VOC), with varying population immune status through vaccinations and prior infections.

The households included at least one member with a laboratory‐confirmed SARS‐CoV‐2 infection and at least two additional household members willing to participate. Recent (< 8 weeks) SARS‐CoV‐2 infection was an exclusion criterion for SL2. Participant characteristics were collected at baseline. All participants, regardless of symptoms, were tested for a SARS‐CoV‐2 infection via (1) reverse transcription polymerase chain reaction (RT‐PCR) on 10 consecutive saliva samples over 6 weeks and (2) IgG antibody titer increase in serum over the same period. For a more detailed description of SARS‐CoV‐2 test methods, see original papers and Supporting Information Materials [8, 9].

All participants included in the two studies were invited to participate in the follow‐up study, completing questionnaires at 6 and 12 months post‐inclusion. Adults completed a persistent symptom questionnaire that was adapted by University Medical Centre (UMC) Groningen (Supporting Information S1: Appendix 1) [10], while children (0–17 years) received a questionnaire developed by Amsterdam UMC and Spaarne Gasthuis (Supporting Information S1: Appendix 2). These questionnaires assessed the presence of symptoms not present before study onset, perceived to be related to SARS‐CoV‐2. Participants who reported persistent symptoms at 6 months were reassessed on persistent symptoms at 12 months.

Additionally, HRQoL and anxiety and depressive symptoms in adults were assessed by the validated 36‐Item Short Form Health Survey (SF‐36) and hospital anxiety and depression scale (HADS), respectively. These questionnaires were administered at 6 and 12 months, regardless of the presence of persistent symptoms [11, 12].

Only participants who completed the 6‐month follow‐up questionnaire were included in our analyses. Participants who reported a SARS‐CoV‐2 (re‐)infection during the 12‐month follow‐up period were excluded from the subsequent follow‐up measurement. Participants who tested negative for SARS‐CoV‐2 during the initial study period were included as control group. Comparison between SARS‐CoV‐2‐positive and ‐negative participants was performed using Fisher's exact test for parametric data and Wilcoxon rank‐sum test for nonparametric data. Finally, possible associations between patient characteristics and persistent symptoms at 6 months were assessed in adults, using univariable logistic regression. A *p* value of less than 0.05 was considered statistically significant. All statistical analyses were conducted using R (version 2023.09.1 + 494). For a more detailed description of the study methods, see Supporting Information.

## 3 Results

**Figure 1:**
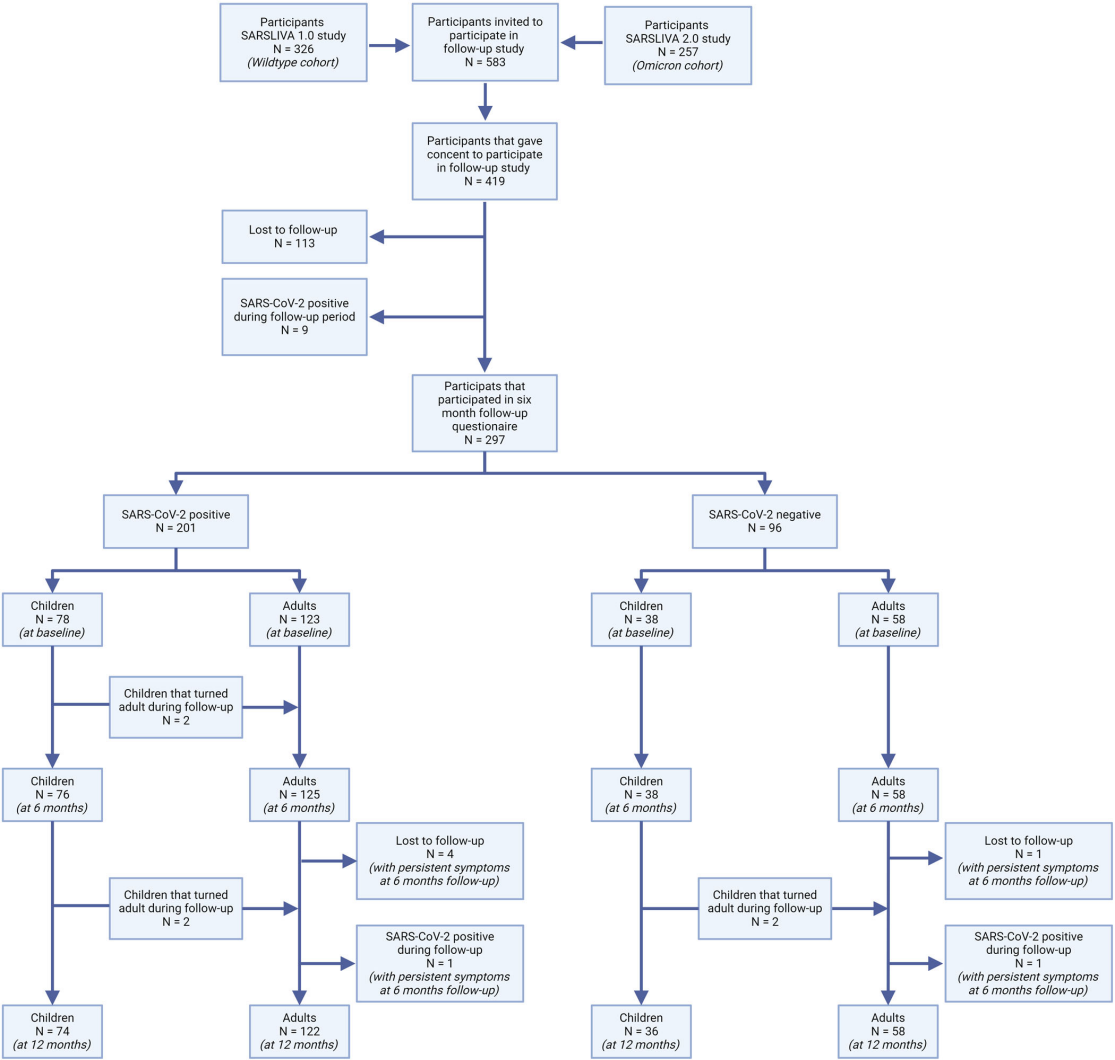
Flowchart of participant selection and consent.

Two hundred and ninety‐seven participants were included, of whom 141 originated from SL1 (50 households) and 156 from SL2 (49 households) (Figure 1). Of the 116 included children, 78 (67.2%) tested positive for SARS‐CoV‐2 (Table 1). Of the 181 included adults, 123 (68.0%) tested positive for SARS‐CoV‐2. Acute symptoms were more frequently reported by SARS‐CoV‐2‐positive participants, such as dyspnea, cough, myalgia, and headache (Table 1). No participants were hospitalized.

**Table 1:** Baseline characteristics.

		SARS‐CoV‐2 infection		
	Overall	Positive	Negative (controls)	*p* value^a^,*
	*N* = 297	*N* = 201	*N* = 96	
Cohort				0.174
SL1 (wild‐type cohort)	141 (47.5%)	101 (50.2%)	40 (41.7%)	
SL2 (Omicron cohort)	156 (52.5%)	100 (49.8%)	56 (58.3%)	
Sex				0.322
Male	136 (45.8%)	88 (43.8%)	48 (50.0%)	
Female	161 (54.2%)	113 (56.2%)	48 (50.0%)	
Age, years	34 (12–48, 0–64)	34 (12–47, 0–61)	28 (12–48, 0–64)	0.863
Age group				0.886
0–11 years old	67 (22.6%)	47 (23.4%)	20 (20.8%)	
12–17 years old	49 (16.5%)	31 (15.4%)	18 (18.8%)	
18–39 years old	56 (18.9%)	40 (19.9%)	16 (16.7%)	
40–49 years old	78 (26.3%)	51 (25.4%)	27 (28.1%)	
50 years and older	47 (15.8%)	32 (15.9%)	15 (15.6%)	
Acute disease severity^b^				< 0.001*
Asymptomatic	84 (28.3%)	33 (16.4%)	51 (53.1%)	
Mild	158 (53.2%)	127 (63.2%)	31 (32.3%)	
Moderately severe	55 (18.5%)	41 (20.4%)	14 (15.6%)	
Immunity children^c^				0.052
Naïve	75 (64.7%)	56 (71.8%)	19 (50.0%)	
Previous infection	21 (18.1%)	13 (16.7%)	8 (21.1%)	
Vaccination	11 (9.5%)	6 (7.7%)	5 (13.2%)	
Hybrid immunity	9 (7.8%)	3 (3.8%)	6 (15.8%)	
Immunity adults				0.006*
Naïve	87 (48.1%)	63 (51.2%)	24 (41.4%)	
Previous infection	1 (0.6%)	1 (0.8%)	0 (0.0%)	
Vaccination	69 (38.1%)	50 (40.7%)	19 (32.8%)	
Hybrid immunity	24 (13.3%)	9 (7.3%)	15 (25.9%)	
BMI class^d^				0.085
Underweight	15 (5.1%)	11 (5.5%)	4 (4.2%)	
Normal weight	186 (62.6%)	117 (58.2%)	69 (71.9%)	
Overweight	74 (24.9%)	54 (26.9%)	20 (20.8%)	
Obesity	22 (7.4%)	19 (9.5%)	3 (3.1%)	
Comorbidities				
Any	108 (36.4%)	75 (37.3%)	33 (34.4%)	0.699
Atopy	80 (26.9%)	55 (27.4%)	25 (26.0%)	0.889
Asthma	17 (5.7%)	12 (6.0%)	5 (5.2%)	1.000
Eczema	26 (8.8%)	20 (10.0%)	6 (6.3%)	0.382
Hay fever	46 (15.5%)	30 (14.9%)	16 (16.7%)	0.733
Dust mite allergy	26 (8.8%)	18 (9.0%)	8 (8.3%)	1.000
Other allergy	14 (4.7%)	11 (5.5%)	3 (3.1%)	0.560
Cardiac	10 (3.4%)	5 (2.5%)	5 (5.2%)	0.302
Pulmonary	2 (0.7%)	2 (1.0%)	0 (0.0%)	1.000
Immunological	2 (0.7%)	2 (1.0%)	0 (0.0%)	1.000
Diabetes mellitus	2 (0.7%)	0 (0.0%)	2 (2.1%)	0.104
Rheumatologic	1 (0.3%)	1 (0.5%)	0 (0.0%)	1.000
Other	25 (8.4%)	20 (10.0%)	5 (5.2%)	0.188
Smoking	7 (2.4%)	5 (2.5%)	2 (2.1%)	1.000
Education (adults)^e^				0.482
Low to normal	60 (37.5%)	40 (35.7%)	20 (41.7%)	
High	100 (62.5%)	72 (64.3%)	28 (58.3%)	
Unknown	21	11	10	
Acute symptoms				
Any	140 (47.1%)	117 (58.2%)	23 (24.0%)	< 0.001*
Cough	93 (31.3%)	76 (37.8%)	17 (17.7%)	< 0.001*
Nasal congestion	117 (39.4%)	97 (48.3%)	20 (20.8%)	< 0.001*
Wheezing	17 (5.7%)	15 (7.5%)	2 (2.1%)	0.067
Dyspnea	36 (12.1%)	31 (15.4%)	5 (5.2%)	0.013*
Painful breathing	9 (3.0%)	8 (4.0%)	1 (1.0%)	0.280
Sore throat	62 (20.9%)	52 (25.9%)	10 (10.4%)	0.002*
Anosmia/ageusia	24 (8.5%)	23 (11.4%)	1 (1.1%)	0.001*
Apnea	2 (0.7%)	2 (1.0%)	0 (0.0%)	1.000
Loss appetite	23 (7.7%)	19 (9.5%)	4 (4.2%)	0.162
Fever	8 (2.7%)	8 (4.0%)	0 (0.0%)	0.058
Vomiting	2 (0.7%)	1 (0.5%)	1 (1.0%)	0.543
Diarrhea	6 (2.0%)	6 (3.0%)	0 (0.0%)	0.182
Headache	81 (27.3%)	67 (33.3%)	14 (14.6%)	< 0.001*
Myalgia	55 (18.5%)	50 (24.9%)	5 (5.2%)	< 0.001*
Other^f^	29 (9.8%)	27 (13.4%)	2 (2.1%)	0.001*

*Note:* Data is presented as *N* (%); median (25%–75%, minimum‐maximum).

Abbreviations: SL1, SARSLIVA 1 study; SL2, SARSLIVA 2 study.

^a^ Comparison between SARS‐CoV‐2‐positive and ‐negative participants using Fisher's exact test for parametric data and the Wilcoxon rank sum test for nonparametric data.

^b^ At baseline, all participants were asked about their disease severity, regardless of SARS‐CoV‐2 diagnosis, simultaneously with obtaining the 10 consecutive saliva samples. Maximal disease severity over these 10 time points was classified as: (1) asymptomatic; (2) mild: some complaints without fever, dyspnea, or hospital admission; or (3) moderate: fever and/or moderate to severe dyspnea without hospital admission.

^c^ Participants were divided based on self‐reported immunity status into naïve, previous infection only, previous vaccination only, and hybrid immunity (both SARS‐CoV‐2 vaccination and previous infection).

^d^ BMI categories for participants < 18 years of age were defined as BMI *z*‐score < −2, underweight; −2 to 1, normal weight; 1–2, overweight; > 2, obesity. (11) BMI categories participants ≥ 18 years of age were defined as < 18.5 kg/m^2^, underweight; 18.5–24.9 kg/m^2^, normal weight; 25.0–29.9 kg/m^2^, overweight; ≥ 30.0 kg/m^2^, obesity.

^e^ Education: (1) High: higher professional education or university education; (2) normal or low: secondary vocational education or lower; reported for adults only.

^f^ Other symptoms: extreme paleness, excessive sweating, temperature intolerance, new hypersensitivities, lymphadenopathy, difficulties chewing or swallowing, difficulties talking, shaking, bladder problems, and other symptoms.

* *p* < 0.05.

From baseline to month 6, two participants reached adult age, resulting in 114 children and 183 adults at 6 months (Figure 1). Of the 114 children, only one reported persistent symptoms at 6 months follow‐up, which were resolved at 12 months follow‐up.

In adults, a larger, though statistically nonsignificant portion of adults of the SARS‐CoV‐2‐positive participants experienced reported persistent symptoms at 6 months follow‐up, compared to the negative participants (16.8% [21/125] vs. 10.3% [6/58]; *p* = 0.370, Table 2). SARS‐CoV‐2‐positive participants reported significantly more persistent respiratory symptoms compared to negative participants (15.2% vs. 3.4%, *p* = 0.023). The most common persistent symptom was dyspnea, which was reported by 10.4% of SARS‐CoV‐2‐positive adults, compared to 3.4% in the negative group (*p* = 0.150). Additionally, a nonsignificant trend for higher prevalence of fatigue was found in the positive group (12.8% vs. 3.4%, *p* 0.061), as for exertion‐related symptoms (8.8% vs. 1.7%, *p* = 0.107).

**Table 2:** Persistent symptoms in adults at 6‐month follow‐up.

	SARS‐CoV‐2 infection		*p* value^a^,*
	Positive (*N* = 125)	Negative (*N* = 58) (controls)	
Any persistent symptoms	21 (16.8%)	6 (10.3%)	0.370
Respiratory symptoms	19 (15.2%)	2 (3.4%)	0.023*
Dyspnea	13 (10.4%)	2 (3.4%)	0.150
Coughing	5 (4.0%)	1 (1.7%)	0.666
Painful breathing	3 (2.4%)	0 (0%)	0.553
Throat pain	2 (1.6%)	1 (1.7%)	1.000
Running nose	4 (3.2%)	1 (1.7%)	1.000
Anosmia	8 (6.4%)	1 (1.7%)	0.276
Ageusia	9 (7.2%)	1 (1.7%)	0.174
Cardiac symptoms	8 (6.4%)	3 (5.2%)	1.000
Palpitations	3 (2.4%)	1 (1.7%)	1.000
Chest pain	7 (5.6%)	2 (3.4%)	0.721
Gastrointestinal symptoms	6 (4.8%)	0 (0%)	0.179
Stomach ache	3 (2.4%)	0 (0%)	0.553
Nausea/vomiting	2 (1.6%)	0 (0%)	1.000
Change in stool	3 (2.4%)	0 (0%)	0.553
Loss of appetite	1 (0.8%)	0 (0%)	1.000
Weight loss	1 (0.8%)	0 (0%)	1.000
Neurocognitive symptoms	12 (9.6%)	3 (5.2%)	0.395
Headache	5 (4.0%)	1 (1.7%)	0.666
Hypersensitivity to light and sound	5 (4.0%)	0 (0%)	0.180
Concentration difficulties	8 (6.4%)	1 (1.7%)	0.276
Memory loss	7 (5.6%)	0 (0%)	0.099
Dizziness	4 (3.2%)	1 (1.7%)	1.000
Balance/coordination problems	5 (4.0%)	2 (3.4%)	1.000
Cognitive impairment	8 (6.4%)	2 (3.4%)	0.508
Double vision	3 (2.4%)	0 (0%)	0.553
Tingling sensation	2 (1.6%)	1 (1.7%)	1.000
Musculoskeletal symptoms	11 (8.8%)	2 (3.4%)	0.232
Muscle complaints	10 (8.0%)	2 (3.4%)	0.344
Joint complaints	6 (4.8%)	1 (1.7%)	0.434
Dermatological symptoms	2 (1.6%)	0 (0%)	1.000
Fever/cold shivers	0 (0%)	0 (0%)	—
Fatigue	16 (12.8%)	2 (3.4%)	0.061
Sleep disturbances	9 (7.2%)	3 (5.2%)	0.755
Exertion‐related symptoms	11 (8.8%)	1 (1.7%)	0.107
Other symptoms	8 (6.4%)	2 (3.4%)	0.508

*Note:* Data is presented as *N* (%).

^a^ Fisher's exact test.

* *p* < 0.05.

At 12 months follow‐up, five participants were lost to follow‐up, and two participants were excluded due to a SARS‐CoV‐2 (re‐)infection during the 12‐month follow‐up period (Figure 1). Of the 122 SARS‐CoV‐2‐positive adults included in the analysis, 9.0% (11/122) reported persistent symptoms at 12 months compared to 5.2% (3/58) of negative participants (*p *= 0.553; Supporting Information S1: Table 1). Respiratory symptoms (7.4% vs. 1.7%), neurocognitive symptoms (7.4% vs. 3.4%), and fatigue (5.7% vs. 1.7%) were most commonly reported, although no statistically significant differences were found in the prevalence of specific persistent symptoms at 12 months follow‐up.

Several factors were found to be statistically significantly associated with the presence of at least one persistent symptom in adults at 6 months (Table 3). The identified factors include obesity (OR 3.82; CI 1.14–12.15), moderate acute disease severity (OR 3.45; CI: 1.07–12.32), a history of eczema (OR 9.25; CI 3.17–27.72), and smoking (OR 26.96; CI 3.79–539.93). Of the acute symptoms, nasal congestion (OR 3.20; CI 1.38–7.90) and dyspnea (OR 3.05; CI 1.18–7.53) were statistically significantly associated with persistent symptoms, as well as a moderately severe acute disease course (OR 3.45; CI 1.07–12.32). The number of symptomatic cases was insufficient to perform risk factor analysis through multivariable analysis (*n* = 27).

**Table 3:** Univariate analysis to test for factors associated with persistent symptoms at 6 months follow‐up in adult participants.

	Persistent symptoms		Univariate logistic regression	
	Yes (*n* = 27)	No (*n* = 156)	OR (95% CI)	*p* value^a^,*
Sex				
Male	13 (48.1%)	71 (45.5%)	Ref.	
Female	14 (51.9%)	85 (54.5%)	0.90 (0.40–2.06)	0.800
Age (median, IQR)	48 (43–52, 17–60)	45 (36–49, 17–64)	1.04 (1.00–1.09)	0.081
SARS‐CoV‐2 test				
Negative (controls)	6 (22.2%)	52 (33.3%)	Ref.	
Positive	21 (77.8%)	104 (66.7%)	1.75 (0.70–5.00)	0.256
SARS‐CoV‐2 variant				
Wildtype	12 (44.4%)	52 (33.3%)	Ref.	
Omicron	9 (33.3%)	52 (33.3%)	0.75 (0.28–1.92)	0.551
Acute disease severity^b^				
Asymptomatic	5 (18.5%)	46 (29.5%)	Ref.	
Mild	13 (48.1%)	86 (55.1%)	1.39 (0.49–4.55)	0.554
Moderate	9 (33.3%)	24 (15.4%)	3.45 (1.07–12.32)	0.043*
Immunity status^c^				
None	15 (55.6%)	73 (46.8%)	Ref.	
Infection	1 (3.7%)	0 (0.0%)	NA	NA
Vaccination	6 (22.2%)	63 (40.4%)	0.46 (0.16–1.21)	0.134
Hybrid	5 (18.5%)	20 (12.8%)	1.22 (0.36–3.58)	0.733
BMI class^d^				
Underweight	0 (0.0%)	5 (3.2%)	0.00	0.989
Normal	11 (40.7%)	84 (53.8%)	Ref.	
Overweight	10 (37.0%)	55 (35.3%)	1.39 (0.54–3.51)	0.485
Obese	6 (22.2%)	12 (7.8%)	3.82 (1.14–12.15)	0.024*
Comorbidities				
Any	15 (55.6%)	52 (33.3%)	1.90 (0.83–4.39)	0.128
Any atopy	12 (44.4%)	43 (27.6%)	2.10 (0.90–4.85)	0.082
Asthma	4 (14.8%)	10 (6.4%)	2.54 (0.65–8.32)	0.141
Eczema	9 (33.3%)	8 (5.1%)	9.25 (3.17–27.72)	< 0.001*
Hay fever	8 (29.6%)	27 (17.3%)	2.01 (0.76–4.95)	0.138
Dust mite allergy	4 (14.8%)	14 (9.0%)	1.76 (0.47–5.43)	0.352
Other allergy	3 (11.1%)	6 (3.8%)	3.13 (0.63–12.71)	0.124
Cardiovascular disease	0 (0.0%)	10 (6.4%)	0.00	0.990
Pulmonary disease	2 (7.4%)	0 (0.0%)	NA	NA
Immune disorder	1 (3.7%)	1 (0.6%)	5.96 (0.23–153.93)	0.212
Diabetes mellitus	0 (0.0%)	2 (1.3%)	0.00	0.989
Rheumatoid disease	0 (0.0%)	0 (0.0%)	NA	NA
Other	2 (7.4%)	15 (9.6%)	0.75 (0.11–2.89)	0.716
Smoking	4 (14.8%)	1 (0.6%)	26.96 (3.79–540)	0.004*
Education^e^				
Low/normal	11 (40.7%)	49 (31.4%)	Ref.	
High	14 (51.9%)	86 (55.1%)	0.73 (0.31–1.75)	0.466
Acute symptoms				
Any	19 (70.4%)	79 (50.6%)	2.31 (0.99–5.90)	0.063
Cough	13 (48.1%)	54 (34.6%)	1.75 (0.76–4.02)	0.181
Nasal congestion	18 (66.7%)	60 (38.5%)	3.20 (1.38–7.90)	0.008*
Wheezing	3 (11.1%)	9 (5.7%)	2.04 (0.43–7.42)	0.309
Dyspnea	9 (33.3%)	22 (14.1%)	3.05 (1.18–7.53)	0.017*
Painful breathing	3 (11.1%)	5 (3.2%)	3.78 (0.74–16.42)	0.082
Throat pain	9 (33.3%)	35 (22.4%)	1.73 (0.69–4.11)	0.225
Anosmia/ageusia	4 (14.8%)	16 (10.3%)	1.61 (0.43–4.94)	0.432
Apnea	0 (0.0%)	1 (0.6%)	0.00	0.992
Loss appetite	3 (11.1%)	9 (5.8%)	2.04 (0.43–7.42)	0.309
Fever	1 (3.7%)	4 (2.6%)	1.46 (0.07–10.37)	0.739
Vomiting	0 (0.0%)	1 (0.6%)	0.00	0.992
Diarrhea	2 (7.4%)	3 (1.9%)	4.08 (0.52–25.82)	0.134
Headache	13 (48.1%)	48 (30.8%)	2.09 (0.90–4.81)	0.081
Muscle ache	11 (40.7%)	35 (22.4%)	2.38 (0.99–5.56)	0.047*
Other^f^	5 (18.5%)	15 (9.6%)	2.14 (0.64–6.16)	0.179

*Note:* Data is presented as *N* (%); median (25%–75%, minimum–maximum).

Abbreviations: CI, confidence interval; OR, odds ratio.

^a^ Univariate logistic regression was used to assess the association between patient characteristics and persistent symptoms in adult participants (*n* = 183).

^b^ At baseline, all participants were asked about their disease severity, simultaneously with obtaining the 10 consecutive saliva samples. Maximal disease severity over these 10 time points was classified as: (1) asymptomatic; (2) mild: some complaints without fever, dyspnea, or hospital admission; or (3) moderate: fever and/or moderate to severe dyspnea without hospital admission.

^c^ Participants were divided based on self‐reported immunity status into naïve, previous infection only, previous vaccination only, and hybrid immunity (both SARS‐CoV‐2 vaccination and previous infection).

^d^ BMI categories for participants < 18 years of age were defined as BMI *z*‐score < −2, underweight; −2 to 1, normal weight; 1–2, overweight; > 2, obesity. (11) BMI categories participants ≥ 18 years of age were defined as < 18.5 kg/m^2^, underweight; 18.5–24.9 kg/m^2^, normal weight; 25.0–29.9 kg/m^2^, overweight; ≥ 30.0 kg/m^2^, obesity.

^e^ Education: (1) High: higher professional education or university education; (2) normal or low: secondary vocational education or lower; reported for adults only.

^f^ Other symptoms: extreme paleness, excessive sweating, temperature intolerance, new hypersensitivities, lymphadenopathy, difficulties chewing or swallowing, difficulties talking, shaking, bladder problems, and other symptoms.

* *p* < 0.05.

In adults, HRQoL, anxiety, and depressive symptom scores did not differ statistically significantly between SARS‐CoV‐2‐positive and ‐negative participants at 6‐ and 12‐month follow‐up (Supporting Information S1: Tables 2 and 3). However, when comparing adults with and without persistent symptoms at 6 months follow‐up, participants with persistent symptoms reported lower HRQoL on most SF‐36 domains, including the physical and mental component scores (PCS and MCS, respectively) and reported a clinically relevant increase in anxiety and depressive symptoms at 6 months follow‐up (Supporting Information S1: Table 4). At 12 months follow‐up, adults with persistent symptoms scored lower, particularly on the physical domains of the SF‐36 and the PCS score (Supporting Information S1: Table 5). The same observations were made in a subgroup analysis comparing SARS‐CoV‐2‐positive adults with and without persistent symptoms (Supporting Information S1: Tables 6 and 7).

## 4 Discussion

This prospective questionnaire study reports on the occurrence of self‐reported persistent symptoms in nonhospitalized children and adults after a SARS‐CoV‐2 infection. By including noninfected household members as a control group, this study ensured a well‐matched comparison, enhancing the reliability and accuracy of the findings through minimized confounding factors. It demonstrates that both infected and noninfected children have a very low prevalence of persistent symptoms. Among adults, the occurrence of at least one symptom was not significantly different between infected and noninfected individuals. However, persistent respiratory symptoms were statistically significantly more often reported among SARS‐CoV‐2‐positive adults than negatively tested adults. Moreover, fatigue and exertion‐related symptoms tend to be more prevalent in SARS‐CoV‐2‐infected adults. Finally, we found that persistent symptoms lead to an impaired HRQoL and increased complaints of anxiety and depression.

The persistence of symptoms for at least 3 months postinfection is commonly used to define post‐acute sequelae of COVID‐19 (PASC), derived from the World Health Association (WHO)‐definition [13], often referred to as long‐COVID. However, the term “long‐COVID” is increasingly viewed as a broad, nonspecific label that encompasses a wide variety of symptoms, potentially oversimplifying the complexity of the condition [14]. Literature highlights that PASC manifests in multiple organ systems, such as respiratory, neurological, and cardiovascular, each with distinct underlying pathophysiological mechanisms [15]. Recent literature therefore emphasizes the importance of focusing on specific symptoms or symptom clusters rather than treating the condition as a singular entity, as this approach may improve understanding and treatment by acknowledging the multifaceted nature of the syndrome [14, 16]. Our findings on differences in specific symptoms align closely with existing literature, which reports a higher prevalence of fatigue, dyspnea, brain fog, and anosmia in nonhospitalized COVID‐19 adults [17]. Notably, our study reinforced these findings within a highly controlled cohort. Not all observed differences in specific symptoms reached statistical significance in this study, possibly due to the limited sample size and associated reduction in statistical power.

This study observed persistent symptoms in the SARS‐CoV‐2‐negative participants, indicating that long‐term symptoms are not exclusively related to SARS‐CoV‐2 infections. Previous research has identified other infectious diseases, including rhinovirus, influenza, respiratory syncytial virus, Epstein−Barr virus, and Lyme disease, as being associated with long‐term symptoms [18, 19, 20, 21, 22, 23]. A recent study further highlighted that individuals with acute respiratory infections (other than SARS‐CoV‐2) had a significantly higher prevalence of persistent symptoms compared to those without respiratory infections, across all COVID‐19 pandemic phases [24]. The current study did not test for viral pathogens other than SARS‐CoV‐2. Yet, the acute symptoms observed in the negative participants suggest that other infections may have contributed to the high prevalence of persistent symptoms in the negative participants, supporting the need for further research into the role of persistent symptoms after (respiratory) infections in general.

The results of the current study demonstrate that adults with persistent symptoms experience a significantly decreased physical and mental HRQoL, alongside a clinically relevant increase in anxiety and depressive symptoms. Recent evidence in over 10 000 adult patients reinforces the profound impact of persistent symptoms. In individuals with post‐COVID symptoms ≥ 3 months after the initial infection, HRQoL remained “poor” up to 24 months after infection, with 22%–32% of participants reporting validated questionnaire scores indicative of anxiety or depressive disorder [25].

Moreover, various studies reported reduced work ability in individuals with persistent symptoms, underscoring the long‐term consequences on functionality and productivity [26, 27], which also contribute to a significant societal burden alongside the increased healthcare utilization and costs associated with these patients [28, 29, 30]. Studies suggest that the type of persistent symptoms strongly influences the degree of individual impairment and societal burden [31, 32], again highlighting the need for a symptom‐specific or symptom‐cluster approach to understand and manage this condition. These findings underscore the substantial disease burden of persistent symptoms after SARS‐CoV‐2 infections at both individual and societal levels, emphasizing the urgent need for comprehensive research to better understand and mitigate these effects.

A key strength of this study lies in its prospective design and comprehensive SARS‐CoV‐2 testing methodology. Another strength is the inclusion of SARS‐CoV‐2‐negative household members as the control group. Limitations include the small sample size, which may have resulted in (1) underdetection of differences in the occurrence of persistent symptoms between the SARS‐CoV‐2‐positive and ‐negative participants, and (2) constraints in our (multivariate) statistical analysis to study risk factors, including pandemic phase. Additionally, recall and selection bias toward participants with persistent symptoms are possible. The lack of a 3‐month follow‐up also prevents reporting the prevalence of post‐COVID condition as defined by the WHO [13]. However, in line with this definition, we specifically inquired about persistent symptoms perceived to be related to the SARS‐CoV‐2 infection. Moreover, despite excluding participants who reported a SARS‐CoV‐2 (re)infection during the follow‐up period, asymptomatic SARS‐CoV‐2 infections cannot be ruled out. We recommend including serological testing at multiple time points in future studies.

In conclusion, this study found that SARS‐CoV‐2‐positive adults tend to have a higher prevalence of persistent fatigue, respiratory, and exertion‐related symptoms as compared to negative household members. Persistent symptoms were associated with a reduced HRQoL and increased anxiety and depression. Furthermore, the prevalence of persistent symptoms after a SARS‐CoV‐2 infection in children in this study was very low. These results underline the importance of incorporating a well‐matched control group in future studies and emphasize the necessity of heightened attention to recovery following infectious diseases in general.

## Author Contributions

Steven F. L. v. Lelyveld, Marianne A. v. Houten, Mildred E. Haverkort, Judith G. C. Sluiter‐Post, Angelique M. A. M. Winkel, Coen R. Lap, and Adriana Tami contributed to the conception and design of the study. Angelique M. A. M. Winkel, Coen R. Lap, and Judith G. C. Sluiter‐Post. participated in the acquisition of data. Dirk Eggink coordinated the laboratory analyses. Angelique M. A. M. Winkel, Bastienne A. d. Jonghe, Steven F. L. v. Lelyveld, and Marianne A. v. Houten were responsible for data analyses and interpretation. Bastienne A. v. Jonghe, Angelique M. A. M. Winkel, Coen R. Lap, Steven F. L. v. Lelyveld, Judith G. C. Sluiter‐Post, Sjoerd M. Euser, Dirk Eggink, and Marianne A. v. Houten, Menno D. d. Jong verified the underlying data. Angelique M. A. M. Winkel, Bastienne A. d. Jonghe, Steven F. L. v. Lelyveld, and Marianne A. Houten wrote the manuscript. All authors had full access to all the data and reviewed and approved the final version of the manuscript.

## Ethics Statement

Written informed consent was obtained from all participants. This study was reviewed and approved by the Medical Ethical Committee of the Amsterdam University Medical Centre, The Netherlands (Reference Numbers 2020.436 [SARSLIVA 1] and 2022.0073 [SARSLIVA 2.0]).

## Conflicts of Interest

The authors declare no conflicts of interest.

## Supporting information

Supplementary_material_Clean.

## Data Availability

Deidentified data collected for the study, including individual participant data collected during the study and a data dictionary, are available from the corresponding author on reasonable request. Requests should be directed to s.van.lelyveld@spaarnegasthuis.nl or mavanhouten@spaarnegasthuis.nl. These requests will be discussed with all project partners (Spaarne Gasthuis, Public Health Services Kennemerland, and RIVM). These requests will be reviewed and approved by the investigator and project partners based on scientific merit. To gain access, data requesters will need to sign a data access agreement. Privacy‐sensitive data, which is traceable to the participant, will not be shared.
