# Supplementary material for: Persistent Symptoms in SARS‐CoV‐2‐Infected and Non‐Infected Household Members: A Prospective Cohort Study
Source: J Med Virol. 2025 Dec 6;97(12):e70727. doi: 10.1002/jmv.70727 (PMC12680964; doi:10.1002/jmv.70727)

**Supplementary Materials**

**Content**

- Supplementary Methods Page 2-4
  - Study design and participant population Page 2
  - Outcomes and measurements Page 2
  - Questionnaires Page 3
  - Statistical analysis Page 4
  - Ethics Page 4
- Supplementary Results Page 5-12
  - Supplementary Table 1 Page 5
  - Supplementary Table 2 Page 6
  - Supplementary Table 3 Page 7
  - Supplementary Table 4 Page 8
  - Supplementary Table 5 Page 9
  - Supplementary Table 6 Page 10
  - Supplementary Table 7 Page 11
- References Page 12
- Supplementary Appendix 1: Long-COVID symptom questionnaire adults Page 13-19
- Supplementary Appendix 2: Long-COVID symptom questionnaire children Page 20-26

**Supplementary methods**

Study design and participant population

This prospective questionnaire study is a 12-month follow-up of the SARSLIVA 1.0 and SARSLIVA 2.0 cohorts, each with a nearly identical study design ^1,2^. These cohorts consist of adults and children from Dutch households aged 0 to 65 years old. The SARSLIVA 1.0 and 2.0 study participants were included from October to December 2020 and March to April 2022 respectively. Households were eligible for inclusion in the initial studies if at least one household member had a laboratory-confirmed SARS-CoV-2 infection through reversed transcription polymerase chain reaction (RT-PCR) on a combined nasopharyngeal swab (NPS) and oropharyngeal swab (OPS). This household member was indicated as the index case. Moreover, at least two additional household members were willing to participate in the study. For the SARSLIVA 1 study,

Over the course of 6 weeks, all participants (both index case and household members) were tested for SARS-CoV-2 on ten consecutive saliva samples using PCR to evaluate household transmission. Saliva samples were obtained by self-sampling at home on days 1, 3, 5, 7, 10, 14, 21, 28, 35, and 42, with the day of inclusion as day 1. The research team collected capillary blood samples during a home visit on day 42 in the SARSLIVA 1 cohort and on days 1 and 42 for the SARSLIVA 2 cohort. Moreover, for the SARSLIVA 1 cohort, NP/OP was performed on day 7. This 6-week follow-up period evaluating the SARS-CoV-2 positivity of household members is referred to as baseline.

*Saliva collection and PCR analysis*

Saliva samples were self-obtained by participants at day 1, 3, 5, 7, 10, 14, 21, 28, 35, and 42 (with day of inclusion as day 1. The participants were instructed to stop drinking, eating, teeth brushing and smoking at least 30 minutes before saliva collection and to avoid clearing their throats or coughing during saliva collection. Saliva was collected using different methods depending on the age of the participants. Participants from the age of five were instructed to spit at least 2 mL of saliva into a Genefix Saliva Collection device without buffer (Isohelix), using funnels to ensure clean collection. Children below the age of five were instructed to place two Oracol (S10) sponges (Malvern Medical Developments) in the mouth for one to two minutes and rub these against the gums, before placing these in a tube. The saliva samples were stored in the participant’s home freezer. At day 42, saliva samples were transported to the laboratory on dry ice for storage at -80 degrees Celsius (°C).

RT-qPCR is performed on the extracted RNA of self-sampled saliva by the laboratory of the National Institute for Public Health and the Environment (RIVM), Bilthoven, the Netherlands. The extraction was executed using MagNApure 96 (MP96) with total nucleic acid kit small volume (Roche). The Roche LC480 II thermal cycler is used for PCR on 5 µl of nucleic acid in TaqMan® Fast Virus 1-Step Master Mix (Thermo Fisher) and SARS-like beta coronavirus (Sarbeco) specific E-gene primers are added and 123 probe and EAV are used as controls, as described earlier.^3^

*Blood collection and protein microarray*

The research team collected capillary blood samples during a home visit on day 42 in the SARSLIVA 1 cohort and on days 1 and 42 for the SARSLIVA 2 cohort. These blood samples were transported by post to the laboratory for serological analysis. The samples were spun down and stored at -80°C upon receival at the laboratory.

Sera were tested for the presence of immunoglobulin G antibodies reactive with the SARS-CoV-2 spike trimer and Nucleocapsid antigens in a protein microarray, in duplicate 2-fold serial dilutions starting at 1:20, essentially as described previously.^4^ For each antigen, a 4-parameter log logistic calibration curve was generated and effective concentration 50, mid-point antibody titers were calculated. Maximal fluorescent signal readout was at 43740 and minimum at 10. Raw data were processed with R version 4.04 statistical software.

*SARS-CoV-2 infection at baseline*

Index cases of both cohorts were included after a laboratory-confirmed SARS-CoV-2 infection was confirmed through reverse transcription polymerase chain reaction (RT-PCR) on a combined nasopharyngeal swab (NPS) and oropharyngeal swab (OPS), as stated before. Therefore, all index cases were SARS-CoV-2-positive at baseline.

SARS-CoV-2 infection of household members at baseline (first 6 weeks) was defined based on RT-PCR results in the saliva samples and on serology. In the SARSLIVA 1 cohort, SARS-CoV-2 infection of household members at baseline was defined as either at least 1 positive SARS-CoV-2 RT-PCR result on 1 of the saliva samples, on NPS or OPS at day 7, or detection of serum antibodies at day 42, regardless of the presence of symptoms. In the SARSLIVA 2 cohort SARS-CoV-2 infection of household members at baseline was defined as a SARS-CoV-2 positive RT-PCR in the saliva of at least one of the ten time points and/or a >5-fold rise of SARS-CoV-2 specific antibodies in serum between day 1 and day 42. Participants without a positive PCR and serology were classified as SARS-CoV-2 negative in both cohorts.

On the saliva samples of SARS-CoV-2 participants with the highest viral load, whole genome sequencing was performed to determine the viral strain. SARS-CoV-2-positive participants included in the SARSLIVA 1.0 study were all infected with the Wild-type variant and participants included in the SARSLIVA 2.0 study were all infected with the Omicron BA.2 variant in accordance with the at that time predominant virus variant. Additionally, data on demographics and symptoms of the acute COVID-19 infection were gathered. Acute disease severity was defined on a three-item scale as (1) asymptomatic; (2) mild: some complaints without fever, dyspnea or hospital admission; or (3) moderate: fever and/or moderate to severe dyspnea without hospital admission. Immunity status was defined based on self-reported information, with participants categorized into four groups: naïve, previous infection only, previous vaccination only, and hybrid immunity (both SARS-CoV-2 vaccination and previous infection).

All participants included in the original two studies, including the participants who did not have a (known) SARS-CoV-2 infection, were asked to participate in the follow-up study. Participants who gave consent for the follow-up study but did not participate in the 6-month follow-up questionnaire were excluded from the analysis. Participants who reported a SARS-CoV-2 (re-)infection during the follow-up period were excluded from the subsequent follow-up measurement. Participants who tested negative for SARS-CoV-2 during the initial study period were included as a control group.

*Saliva (self-)sampling*

Multiple studies have demonstrated a comparable or higher sensitivity of detecting SARS-CoV-2 using saliva compared to NPS, both in adults and children ^5–9^. Furthermore, RT-PCR on saliva effectively identifies SARS-CoV-2 even before the period of infectiousness ^10^, and particularly high sensitivity rates of RT-PCR on saliva have been found among studies involving asymptomatic individuals ^11,12^. Compared to NPS, salivary testing is less invasive, and samples can be easily collected by individuals themselves (including children with the assistance of their parents) without the need for qualified personnel, allowing frequent sampling. Moreover, salivary self-sampling has been shown to be more sensitive than self-administered nasal swabs ^13^.

Outcomes and measurements

The primary outcome measure was the prevalence of persistent symptoms 6 and 12 months after non-hospitalized SARS-CoV-2 infection compared to uninfected individuals.

Subsequently, we compared the prevalence between the two different phases of the COVID-19 pandemic to assess whether there is an association between phase and risk of persistent symptoms. To assess persistent symptoms, adult participants completed the Long-COVID questionnaire (“Lange COVID vragenlijst” in appendix 1) at 6 and 12 months. This is a general questionnaire on persistent symptoms after a SARSCoV-2 infection developed by University Medical Centre (UMC) Groningen (Appendix 1). The main difference between these questionnaires is that the pediatric questionnaire is shorter since it does not ask about the severity of each symptom. Moreover, limitations for school are assessed instead of limitations for work. Children who turned 18 during the follow-up period would receive an adult questionnaire at the subsequent follow-up measurement. All participants were asked about the presence of persistent symptoms related to SARS-CoV-2 at 6 months follow-up. Only if they reported to have symptoms at 6 months that were not present before the start of the study and were perceived to be related to SARS-CoV-2, a more detailed set of questions about specific symptoms followed. If participants reported persistent symptoms at 6 months follow-up, they were asked about persistent symptoms at 12 months. Therefore, we aimed to minimize the reporting of symptoms that started more than 6 months following the infection. Due to expanded knowledge of symptomology over the course of the COVID-19 pandemic, there were slight differences in the questionnaires between the SARSLIVA 1.0 and 2.0 studies. For analysis, these were aligned as much as possible (Appendix 3).

Two secondary outcome measures of interest were defined. First, HRQoL, anxiety and depressive symptoms in adult participants with persistent symptoms compared to participants without these. For this purpose, validated questionnaires were used. The 36-Item Short Form Health Survey (SF-36) was used to assess health-related quality of life and the Hospital Anxiety and Depression Scale (HADS) was used to measure symptoms of anxiety and depression. These questionnaires were sent to all adults at 6 and 12 months follow-up, regardless of the presence of persistent symptoms. Second, possible associations between patient characteristics and persistent symptoms were assessed using logistic regression.

Questionnaires

*36-Item Short Form Health Survey*

The 36-Item Short Form Health Survey (SF-36) measures health-related quality of life in adults on nine domains: physical functioning, role limitations due to physical health, bodily pain, energy/fatigue, social functioning, role limitations due to emotional health, emotional well-being and general health ^14,15^. Scores range from 0 to 100, and a higher score indicates better quality of life. Additionally, two summary scores can be calculated, the physical component score (PCS) and the mental component score (MSC). These scores are standardized using normative data from the 1998 United States general population. The mean score is 50 with a standard deviation of 10, a higher score indicates better health-related quality of life. A minimal clinically important difference (MCID) has not been defined for post-COVID condition yet.

*Hospital Anxiety and Depression Scale*

The Hospital Anxiety and Depression Scale (HADS) measures complaints of anxiety and depression in adults over the previous four weeks ^16,17^ The questionnaire consists of 14 questions, 7 about anxiety and depression each. A total score for the domains of depression and anxiety is calculated. Scores range from 0 to 21; a higher score indicates more complaints. The cut-off point for clinically relevant anxiety or depressive symptoms is 8 points.

Statistical analysis

Categorical variables were reported as total count and percentages. Not-normally distributed continuous variables were reported as median, interquartile range and minimum and maximum range. For comparability with existing literature, the scores of the SF-36 questionnaire are presented as mean with standard deviation. Significance testing for categorical variables was done by Fisher’s exact test. We used the Wilcoxon rank sum test for continuous variables, if not normally distributed. Logistic regression analysis was performed to calculate odds ratios (OR) with 95% confidence intervals (CI) for the occurrence of persistent symptoms. We did not correct for multiple comparisons. A p-value of less than 0.05 was considered statistically significant. All statistical analyses were conducted using R (version 2023.09.1+494).

Ethics

The SARSLIVA 1.0 and 2.0 study, including the follow-up amendment, were approved by the Medical Ethics Committee of VU Medical Centre (ref. nr. NL74555.029.20) and the Medical Ethics Committee of Amsterdam UMC (ref. nr. NL80523.029.22) respectively. Written informed consent was obtained.

**Supplementary results**

| *Supplementary Table 1. Persistent symptoms in adults at 12 months follow-up* | | | |
| --- | --- | --- | --- |
|  | **SARS-CoV-2 infection** | | **p-value*^2^*** |
|  | **Positive**, N = 122*^1^* | **Negative**, N = 58*^1^* |  |
| Any persistent symptoms | 11 (9.0%) | 3 (5.2%) | 0.553 |
| Respiratory symptoms | 9 (7.4%) | 1 (1.7%) | 0.171 |
| Dyspnoea | 6 (4.9%) | 1 (1.7%) | 0.431 |
| Coughing | 5 (4.1%) | 0 (0%) | 0.117 |
| Painful breathing | 2 (1.6%) | 0 (0%) | 1.000 |
| Throat pain | 1 (0.8%) | 0 (0%) | 1.000 |
| Running nose | 2 (1.6%) | 0 (0%) | 1.000 |
| Anosmia | 4 (3.3%) | 0 (0%) | 0.307 |
| Ageusia | 5 (4.1%) | 0 (0%) | 0.177 |
| Cardiac symptoms | 4 (3.3%) | 0 (0%) | 0.307 |
| Palpitations | 2 (1.6%) | 0 (0%) | 0.552 |
| Chest pain | 2 (1.6%) | 0 (0%) | 1.000 |
| Gastrointestinal symptoms | 4 (3.3%) | 0 (0%) | 0.307 |
| Stomach ache | 2 (1.6%) | 0 (0%) | 1.000 |
| Nausea/vomiting | 1 (0.8%) | 0 (0%) | 1.000 |
| Change in stool | 3 (2.4%) | 0 (0%) | 0.552 |
| Loss of appetite | 1 (0.8%) | 0 (0%) | 1.000 |
| Weight loss | 1 (0.8%) | 0 (0%) | 1.000 |
| Neurocognitive symptoms | 9 (7.4%) | 2 (3.4%) | 0.507 |
| Headache | 3 (2.5%) | 1 (1.7%) | 1.000 |
| Hypersensitivity to light and sound | 3 (2.5%) | 0 (0%) | 0.552 |
| Concentration difficulties | 5 (4.1%) | 0 (0%) | 0.177 |
| Memory loss | 6 (4.9%) | 0 (0%) | 0.179 |
| Dizziness | 1 (0.8%) | 0 (0%) | 1.000 |
| Balance/coordination problems | 4 (3.3%) | 0 (0%) | 0.307 |
| Cognitive impairment | 6 (4.9%) | 0 (0%) | 0.179 |
| Double vision | 1 (0.8%) | 0 (0%) | 1.000 |
| Tingling sensation | 1 (0.8%) | 2 (3.4%) | 0.243 |
| Musculoskeletal symptoms | 5 (4.1%) | 1 (1.7%) | 0.666 |
| Muscle complaints | 3 (2.5%) | 1 (1.7%) | 1.000 |
| Joint complaints | 4 (3.3%) | 0 (0%) | 0.307 |
| Dermatological symptoms | 3 (2.5%) | 0 (0%) | 0.552 |
| Fever/cold shivers | 0 (0%) | 0 (0%) | - |
| Fatigue | 7 (5.7%) | 1 (1.7%) | 0.440 |
| Sleep disturbances | 4 (3.3%) | 2 (3.4%) | 1.000 |
| Exertion related symptoms | 4 (3.3%) | 0 (0%) | 0.307 |
| Other symptoms | 4 (3.3%) | 1 (1.7%) | 1.000 |
| ^1^ n (%)  ^2^ Fisher’s exact test | | | |

| *Supplementary Table 2. Quality of life of all adults at 6 months follow-up* | | | |
| --- | --- | --- | --- |
|  | **SARS-CoV-2 infection** | | **p-value*^1^*** |
|  | **Positive**, N = 125*^1^* | **Negative**, N = 58*^1^* |  |
| SF-36 |  |  |  |
| Physical Functioning |  |  | 0.358 |
| *Mean (SD)* | 97 (8) | 99 (3) |  |
| *Unknown, n (%)* | 3 (2.4%) | 0 |  |
| Role-Physical |  |  | 0.212 |
| *Mean (SD)* | 94 (22) | 99 (7) |  |
| *Unknown, n (%)* | 4 (3.2%) | 0 |  |
| Bodily Pain |  |  | 0.693 |
| *Mean (SD)* | 93 (14) | 94 (13) |  |
| *Unknown, n (%)* | 3 (2.4%) | 0 |  |
| General Health |  |  | 0.660 |
| *Mean (SD)* | 79 (15) | 79 (13) |  |
| *Unknown, n (%)* | 3 (2.4%) | 0 |  |
| Vitality |  |  | 0.819 |
| *Mean (SD)* | 75 (16) | 76 (15) |  |
| *Unknown, n (%)* | 4 (3.2%) | 2 (3.4%) |  |
| Social Functioning |  |  | 0.538 |
| *Mean (SD)* | 94 (13) | 94 (12) |  |
| *Unknown, n (%)* | 3 (2.4%) | 0 |  |
| Role-Emotional |  |  | 0.967 |
| *Mean (SD)* | 93 (22) | 95 (17) |  |
| *Unknown, n (%)* | 3 (2.4%) | 0 |  |
| Mental Health |  |  | 0.484 |
| *Mean (SD)* | 84 (11) | 82 (13) |  |
| *Unknown, n (%)* | 3 (2.4%) | 0 |  |
| Reported Health Transition |  |  | 0.857 |
| *Mean (SD)* | 51 (15) | 50 (12) |  |
| *Unknown, n (%)* | 3 (2.4%) | 1 (1.7%) |  |
| Physical component score |  |  | 0.406 |
| *Mean (SD)* | 55 (5) | 56 (3) |  |
| *Unknown, n (%)* | 5 (4.0%) | 2 (3.4%) |  |
| Mental component score |  |  | 0.522 |
| *Mean (SD)* | 54 (6) | 54 (7) |  |
| *Unknown, n (%)* | 5 (4.0%) | 2 (3.4%) |  |
| HADS Anxiety |  |  |  |
| Median (IQR) | 3 (3) | 4 (3) | 0.075 |
| Exceeded cut-off score*, n (%)* | 5 (4.2%) | 5 (8.9%) | 0.293 |
| *Unknown, n (%)* | 5 (4.0%) | 2 (3.4%) |  |
| HADS Depression |  |  |  |
| Median (IQR) | 2 (2) | 2 (2) | 0.984 |
| Exceeded cut-off score*, n (%)* | 4 (3.3%) | 2 (3.6%) | 1.000 |
| *Unknown, n (%)* | 5 (4.0%) | 2 (3.4%) |  |
| *^1^* Wilcoxon rank sum test; Fisher’s exact test | | | |

| *Supplementary Table 3. Quality of life of all adults at 12 months follow-up* | | | |
| --- | --- | --- | --- |
|  | **SARS-CoV-2 infection** | | **p-value*^1^*** |
|  | **Positive**, N = 122 | **Negative**, N = 58 |  |
| SF-36 |  |  |  |
| Physical Functioning |  |  | 0.728 |
| *Mean (SD)* | 97 (8) | 97 (6) |  |
| *Unknown, n (%)* | 22 (18.0%) | 12 (20.7%) |  |
| Role-Physical |  |  | 0.871 |
| *Mean (SD)* | 92 (26) | 91 (27) |  |
| *Unknown, n (%)* | 22 (18.0%) | 12 (20.7%) |  |
| Bodily Pain |  |  | 0.243 |
| *Mean (SD)* | 92 (15) | 90 (18) |  |
| *Unknown, n (%)* | 22 (18.0%) | 12 (20.7%) |  |
| General Health |  |  | 0.730 |
| *Mean (SD)* | 77 (15) | 77 (15) |  |
| *Unknown, n (%)* | 22 (18.0%) | 12 (20.7%) |  |
| Vitality |  |  | 0.897 |
| *Mean (SD)* | 75 (15) | 76 (14) |  |
| *Unknown, n (%)* | 22 (18.0%) | 12 (20.7%) |  |
| Social Functioning |  |  | 0.745 |
| *Mean (SD)* | 93 (12) | 91 (16) |  |
| *Unknown, n (%)* | 22 (18.0%) | 12 (20.7%) |  |
| Role-Emotional |  |  | 0.463 |
| *Mean (SD)* | 93 (22) | 90 (25) |  |
| *Unknown, n (%)* | 22 (18.0%) | 12 (20.7%) |  |
| Mental Health |  |  | 0.093 |
| *Mean (SD)* | 85 (11) | 82 (12) |  |
| *Unknown, n (%)* | 22 (18.0%) | 12 (20.7%) |  |
| Reported Health Transition |  |  | 0.747 |
| *Mean (SD)* | 52 (16) | 51 (15) |  |
| *Unknown, n (%)* | 22 (18.0%) | 12 (20.7%) |  |
| Physical component score |  |  | 0.657 |
| *Mean (SD)* | 55 (6) | 55 (5) |  |
| *Unknown, n (%)* | 22 (18.0%) | 12 (20.7%) |  |
| Mental component score |  |  | 0.184 |
| *Mean (SD)* | 55 (6) | 53 (7) |  |
| *Unknown, n (%)* | 22 (18.0%) | 12 (20.7%) |  |
| HADS Anxiety |  |  |  |
| Median (IQR) | 3 (3) | 3 (3) | 0.425 |
| Exceeded cut-off score (n =) | 9 (9.3%) | 5 (10.9%) | 0.769 |
| *Unknown, n (%)* | 25 (20.5%) | 12 (20.7%) |  |
| HADS Depression |  |  |  |
| Median (IQR) | 2 (2) | 2 (2) | 0.859 |
| Exceeded cut-off score (n =) | 4 (4.1%) | 2 (4.3%) | 1.000 |
| *Unknown, n (%)* | 25 (20.5%) | 12 (20.7%) |  |
| *^1^* Wilcoxon rank sum test; Fisher’s exact test | | | |

| *Supplementary Table 4. Quality of life of all adults at 6 months follow-up* | | | |
| --- | --- | --- | --- |
|  | **Persistent symptoms** | | **p-value*^1*^*** |
|  | **Yes**, N = 27 | **No**, N = 156 |  |
| SF-36 |  |  |  |
| Physical Functioning |  |  | **<0.001*** |
| *Mean (SD)* | 90 (13) | 99 (3) |  |
| *Unknown, n (%)* | 0 | 3 (1.9%) |  |
| Role-Physical |  |  | **<0.001*** |
| *Mean (SD)* | 77 (37) | 99 (10) |  |
| *Unknown, n (%)* | 0 | 4 (2.6%) |  |
| Bodily Pain |  |  | **<0.001*** |
| *Mean (SD)* | 82 (22) | 95 (10) |  |
| *Unknown, n (%)* | 0 | 3 (1.9%) |  |
| General Health |  |  | **0.011*** |
| *Mean (SD)* | 70 (21) | 81 (13) |  |
| *Unknown, n (%)* | 0 | 3 (1.9%) |  |
| Vitality |  |  | **<0.001*** |
| *Mean (SD)* | 61 (17) | 78 (14) |  |
| *Unknown, n (%)* | 0 | 6 (3.8%) |  |
| Social Functioning |  |  | **<0.001*** |
| *Mean (SD)* | 81 (21) | 96 (9) |  |
| *Unknown, n (%)* | 0 | 3 (1.9%) |  |
| Role-Emotional |  |  | 0.295 |
| *Mean (SD)* | 86 (34) | 95 (17) |  |
| *Unknown, n (%)* | 0 | 3 (1.9%) |  |
| Mental Health |  |  | **0.003*** |
| *Mean (SD)* | 76 (13) | 84 (11) |  |
| *Unknown, n (%)* | 0 | 3 (1.9%) |  |
| Reported Health Transition |  |  | **<0.001*** |
| *Mean (SD)* | 39 (23) | 53 (11) |  |
| *Unknown, n (%)* | 0 | 4 (2.6%) |  |
| Physical component score |  |  | **<0.001*** |
| *Mean (SD)* | 51 (8) | 56 (3) |  |
| *Unknown, n (%)* | 0 | 7 (4.5%) |  |
| Mental component score |  |  | **0.003*** |
| *Mean (SD)* | 50 (8) | 55 (6) |  |
| *Unknown, n (%)* | 0 | 7 (4.5%) |  |
| HADS Anxiety |  |  |  |
| Median (IQR) | 6 (4) | 3 (2) | **<0.001*** |
| Exceeded cut-off score (n =) | 7 (26.9%) | 3 (2.0%) | **<0.001*** |
| *Unknown, n (%)* | 1 (3.7%) | 6 (3.8%) |  |
| HADS Depression |  |  |  |
| Median (IQR) | 4 (3) | 1 (2) | **<0.001*** |
| Exceeded cut-off score (n =) | 3 (11.5%) | 3 (2.0%) | **0.043*** |
| *Unknown, n (%)* | 1 (3.7%) | 6 (3.8%) |  |
| *^1^* Wilcoxon rank sum test; Fisher’s exact test  ^*^ p < 0.05 | | | |

| *Supplementary Table 5. Quality of life of all adults at 12 months follow-up* | | | |
| --- | --- | --- | --- |
|  | **Persistent symptoms** | | **p-value*^1*^*** |
|  | **Yes**, N = 14 | **No**, N = 166 |  |
| SF-36 |  |  |  |
| Physical Functioning |  |  | **<0.001*** |
| *Mean (SD)* | 88 (14) | 98 (6) |  |
| *Unknown, n (%)* | 1 (7.1%) | 33 (19.9%) |  |
| Role-Physical |  |  | **<0.001*** |
| *Mean (SD)* | 52 (47) | 95 (19) |  |
| *Unknown, n (%)* | 1 (7.1%) | 33 (19.9%) |  |
| Bodily Pain |  |  | **0.004*** |
| *Mean (SD)* | 77 (24) | 93 (14) |  |
| *Unknown, n (%)* | 1 (7.1%) | 33 (19.9%) |  |
| General Health |  |  | **0.014*** |
| *Mean (SD)* | 67 (15) | 78 (15) |  |
| *Unknown, n (%)* | 1 (7.1%) | 33 (19.9%) |  |
| Vitality |  |  | **0.005*** |
| *Mean (SD)* | 64 (14) | 76 (15) |  |
| *Unknown, n (%)* | 1 (7.1%) | 33 (19.9%) |  |
| Social Functioning |  |  | **0.004*** |
| *Mean (SD)* | 82 (20) | 94 (12) |  |
| *Unknown, n (%)* | 1 (7.1%) | 33 (19.9%) |  |
| Role-Emotional |  |  | **0.016*** |
| *Mean (SD)* | 69 (48) | 94 (18) |  |
| *Unknown, n (%)* | 1 (7.1%) | 33 (19.9%) |  |
| Mental Health |  |  | 0.056 |
| *Mean (SD)* | 78 (11) | 84 (11) |  |
| *Unknown, n (%)* | 1 (7.1%) | 33 (19.9%) |  |
| Reported Health Transition |  |  | 0.204 |
| *Mean (SD)* | 48 (24) | 52 (15) |  |
| *Unknown, n (%)* | 1 (7.1%) | 33 (19.9%) |  |
| Physical component score |  |  | **0.007*** |
| *Mean (SD)* | 47 (9) | 55 (5) |  |
| *Unknown, n (%)* | 1 (7.1%) | 33 (19.9%) |  |
| Mental component score |  |  | 0.151 |
| *Mean (SD)* | 50 (9) | 55 (6) |  |
| *Unknown, n (%)* | 1 (7.1%) | 33 (19.9%) |  |
| HADS Anxiety |  |  |  |
| Median (IQR) | 5 (3) | 3 (3) | **0.022*** |
| Exceeded cut-off score (n =) | 2 (15.4%) | 12 (9.2%) | 0.617 |
| *Unknown, n (%)* | 1 (7.1%) | 36 (21.7%) |  |
| HADS Depression |  |  |  |
| Median (IQR) | 4 (2) | 2 (2) | **<0.001*** |
| Exceeded cut-off score (n =) | 1 (7.7%) | 5 (3.8%) | 0.442 |
| *Unknown, n (%)* | 1 (7.1%) | 36 (21.7%) |  |
| *^1^* Wilcoxon rank sum test; Fisher’s exact test  ^*^ p < 0.05 | | | |

| *Supplementary Table 6. Quality of life of all SARS-CoV-2 positive adults at 6 months follow-up* | | | |
| --- | --- | --- | --- |
|  | **Persistent symptoms** | | **p-value*^1*^*** |
|  | **Yes**, N = 21 | **No**, N = 104 |  |
| SF-36 |  |  |  |
| Physical Functioning |  |  | **<0.001*** |
| *Mean (SD)* | 89 (14) | 99 (4) |  |
| *Unknown, n (%)* | 0 | 3 (2.9%) |  |
| Role-Physical |  |  | **<0.001*** |
| *Mean (SD)* | 73 (40) | 98 (12) |  |
| *Unknown, n (%)* | 0 | 4 (3.8%) |  |
| Bodily Pain |  |  | **<0.001*** |
| *Mean (SD)* | 80 (23) | 95 (9) |  |
| *Unknown, n (%)* | 0 | 3 (2.9%) |  |
| General Health |  |  | **0.020*** |
| *Mean (SD)* | 70 (21) | 81 (13) |  |
| *Unknown, n (%)* | 0 | 3 (2.9%) |  |
| Vitality |  |  | **<0.001*** |
| *Mean (SD)* | 60 (19) | 78 (13) |  |
| *Unknown, n (%)* | 0 | 4 (3.8%) |  |
| Social Functioning |  |  | **<0.001*** |
| *Mean (SD)* | 81 (23) | 97 (8) |  |
| *Unknown, n (%)* | 0 | 3 (2.9%) |  |
| Role-Emotional |  |  | 0.093 |
| *Mean (SD)* | 83 (37) | 96 (17) |  |
| *Unknown, n (%)* | 0 | 3 (2.9%) |  |
| Mental Health |  |  | **0.022*** |
| *Mean (SD)* | 77 (14) | 85 (10) |  |
| *Unknown, n (%)* | 0 | 3 (2.9%) |  |
| Reported Health Transition |  |  | **<0.001*** |
| *Mean (SD)* | 38 (23) | 53 (12) |  |
| *Unknown, n (%)* | 0 | 3 (2.9%) |  |
| Physical component score |  |  | **<0.001*** |
| *Mean (SD)* | 50 (8) | 56 (3) |  |
| *Unknown, n (%)* | 0 | 5 (4.8%) |  |
| Mental component score |  |  | **0.020*** |
| *Mean (SD)* | 50 (9) | 55 (5) |  |
| *Unknown, n (%)* | 0 | 5 (4.8%) |  |
| HADS Anxiety |  |  |  |
| Median (IQR) | 5 (4) | 2 (2) | **0.001*** |
| Exceeded cut-off score (n =) | 4 (20.0%) | 1 (1.0%) | **0.003*** |
| *Unknown, n (%)* | 1 (4.8%) | 4 (3.8%) |  |
| HADS Depression |  |  |  |
| Median (IQR) | 4 (3) | 1 (1) | **<0.001*** |
| Exceeded cut-off score (n =) | 3 (15.0%) | 1 (1.0%) | **0.014*** |
| *Unknown, n (%)* | 1 (4.8%) | 4 (3.8%) |  |
| *^1^* Wilcoxon rank sum test; Fisher’s exact test  ^*^ p < 0.05 | | | |

| *Supplementary Table 7. Quality of life of all SARS-CoV-2 positive adults at 12 months follow-up* | | | |
| --- | --- | --- | --- |
|  | **Persistent symptoms** | | **p-value*^1*^*** |
|  | **Yes**, N = 11 | **No**, N = 111 |  |
| SF-36 |  |  |  |
| Physical Functioning |  |  | **<0.001*** |
| *Mean (SD)* | 85 (15) | 98 (5) |  |
| *Unknown, n (%)* | 1 (9.1%) | 21 (18.9%) |  |
| Role-Physical |  |  | **<0.001*** |
| *Mean (SD)* | 48 (46) | 96 (17) |  |
| *Unknown, n (%)* | 1 (9.1%) | 21 (18.9%) |  |
| Bodily Pain |  |  | **0.004*** |
| *Mean (SD)* | 75 (26) | 94 (12) |  |
| *Unknown, n (%)* | 1 (9.1%) | 21 (18.9%) |  |
| General Health |  |  | **0.012*** |
| *Mean (SD)* | 65 (16) | 79 (15) |  |
| *Unknown, n (%)* | 1 (9.1%) | 21 (18.9%) |  |
| Vitality |  |  | **0.004*** |
| *Mean (SD)* | 61 (14) | 76 (15) |  |
| *Unknown, n (%)* | 1 (9.1%) | 21 (18.9%) |  |
| Social Functioning |  |  | **0.001*** |
| *Mean (SD)* | 80 (20) | 95 (10) |  |
| *Unknown, n (%)* | 1 (9.1%) | 21 (18.9%) |  |
| Role-Emotional |  |  | **<0.001** |
| *Mean (SD)* | 60 (52) | 97 (12) |  |
| *Unknown, n (%)* | 1 (9.1%) | 21 |  |
| Mental Health |  |  | 0.105 |
| *Mean (SD)* | 79 (11) | 85 (11) |  |
| *Unknown, n (%)* | 1 (9.1%) | 21 (18.9%) |  |
| Reported Health Transition |  |  | 0.439 |
| *Mean (SD)* | 50 (26) | 52 (15) |  |
| *Unknown, n (%)* | 1 (9.1%) | 21 (18.9%) |  |
| Physical component score |  |  | **0.014*** |
| *Mean (SD)* | 46 (9) | 55 (5) |  |
| *Unknown* | 1 (9.1%) | 21 (18.9%) |  |
| Mental component score |  |  | 0.163 |
| *Mean (SD)* | 49 (10) | 55 (5) |  |
| *Unknown, n (%)* | 1 (9.1%) | 21 (18.9%) |  |
| HADS Anxiety |  |  |  |
| Median (IQR) | 5 (4) | 3 (3) | **0.041*** |
| Exceeded cut-off score (n =) | 2 (20.0%) | 7 (80%) | 0.232 |
| *Unknown, n (%)* | 1 (9.1%) | 24 (21.6%) |  |
| HADS Depression |  |  |  |
| Median (IQR) | 3 (2) | 1 (2) | **<0.001*** |
| Exceeded cut-off score (n =) | 0 (0.0%) | 4 (4.6%) | 1.000 |
| *Unknown, n (%)* | 1 (9.1%) | 24 (21.6%) |  |
| *^1^* Wilcoxon rank sum test; Fisher’s exact test  ^*^ p < 0.05 | | | |

**References**

1. Kolodziej LM, van Lelyveld SFL, Haverkort ME, et al. High Severe Acute Respiratory Syndrome Coronavirus 2 (SARS-CoV-2) Household Transmission Rates Detected by Dense Saliva Sampling. *Clinical Infectious Diseases*. 2022;75(1):e10-e19. doi:10.1093/cid/ciac261

2. Winkel A, Kozanli E, Haverkort ME, et al. Lower levels of household transmission of SARS-CoV-2 VOC Omicron compared to Wild-type: an interplay between transmissibility and immune status. *medRxiv*. Published online January 1, 2024:2024.07.16.24310515. doi:10.1101/2024.07.16.24310515

3. Kolodziej LM, van Lelyveld SFL, Haverkort ME, et al. High Severe Acute Respiratory Syndrome Coronavirus 2 (SARS-CoV-2) Household Transmission Rates Detected by Dense Saliva Sampling. *Clin Infect Dis*. 2022;75(1):e10-e19. doi:10.1093/cid/ciac261

4. van Tol S, Mögling R, Li W, et al. Accurate serology for SARS-CoV-2 and common human coronaviruses using a multiplex approach. *Emerg Microbes Infect*. 2020;9(1):1965-1973. doi:10.1080/22221751.2020.1813636

5. Teo AKJ, Choudhury Y, Tan IB, et al. Saliva is more sensitive than nasopharyngeal or nasal swabs for diagnosis of asymptomatic and mild COVID-19 infection. *Sci Rep*. 2021;11(1):3134. doi:10.1038/s41598-021-82787-z

6. Wyllie AL, Fournier J, Casanovas-Massana A, et al. Saliva or Nasopharyngeal Swab Specimens for Detection of SARS-CoV-2. *New England Journal of Medicine*. 2020;383(13):1283-1286. doi:10.1056/NEJMc2016359

7. Fougère Y, Schwob JM, Miauton A, et al. Performance of RT-PCR on Saliva Specimens Compared With Nasopharyngeal Swabs for the Detection of SARS-CoV-2 in Children. *Pediatric Infectious Disease Journal*. 2021;40(8):e300-e304. doi:10.1097/INF.0000000000003198

8. Bastos ML, Perlman-Arrow S, Menzies D, Campbell JR. The Sensitivity and Costs of Testing for SARS-CoV-2 Infection With Saliva Versus Nasopharyngeal Swabs. *Ann Intern Med*. 2021;174(4):501-510. doi:10.7326/M20-6569

9. Butler-Laporte G, Lawandi A, Schiller I, et al. Comparison of Saliva and Nasopharyngeal Swab Nucleic Acid Amplification Testing for Detection of SARS-CoV-2. *JAMA Intern Med*. 2021;181(3):353. doi:10.1001/jamainternmed.2020.8876

10. Smith RL, Gibson LL, Martinez PP, et al. Longitudinal Assessment of Diagnostic Test Performance Over the Course of Acute SARS-CoV-2 Infection. *J Infect Dis*. 2021;224(6):976-982. doi:10.1093/infdis/jiab337

11. Rao M, Rashid FA, Sabri FSAH, et al. Comparing Nasopharyngeal Swab and Early Morning Saliva for the Identification of Severe Acute Respiratory Syndrome Coronavirus 2 (SARS-CoV-2). *Clinical Infectious Diseases*. 2021;72(9):e352-e356. doi:10.1093/cid/ciaa1156

12. Herrera LA, Hidalgo-Miranda A, Reynoso-Noverón N, et al. Saliva is a reliable and accessible source for the detection of SARS-CoV-2. *International Journal of Infectious Diseases*. 2021;105:83-90. doi:10.1016/j.ijid.2021.02.009

13. Teo AKJ, Choudhury Y, Tan IB, et al. Saliva is more sensitive than nasopharyngeal or nasal swabs for diagnosis of asymptomatic and mild COVID-19 infection. *Sci Rep*. 2021;11(1):3134. doi:10.1038/s41598-021-82787-z

14. 36-Item Short Form Survey (SF-36) | RAND. Accessed May 17, 2024. https://www.rand.org/health-care/surveys_tools/mos/36-item-short-form.html

15. Aaronson NK, Muller M, Cohen PDA, et al. Translation, Validation, and Norming of the Dutch Language Version of the SF-36 Health Survey in Community and Chronic Disease Populations. *J Clin Epidemiol*. 1998;51(11):1055-1068. doi:10.1016/S0895-4356(98)00097-3

16. Hospital Anxiety and Depression Scale – Meetinstrumenten in de zorg. Accessed May 17, 2024. https://meetinstrumentenzorg.nl/instrumenten/hospital-anxiety-and-depression-scale/

17. Spinhoven P, Ormel J, Sloekers PP, Kempen GI, Speckens AE, Van Hemert AM. A validation study of the Hospital Anxiety and Depression Scale (HADS) in different groups of Dutch subjects. *Psychol Med*. 1997;27(2):363-370. doi:10.1017/S0033291796004382

**Supplementary Appendix 1: Persistent symptoms questionnaire adults: “Lange COVID vragenlijst”**


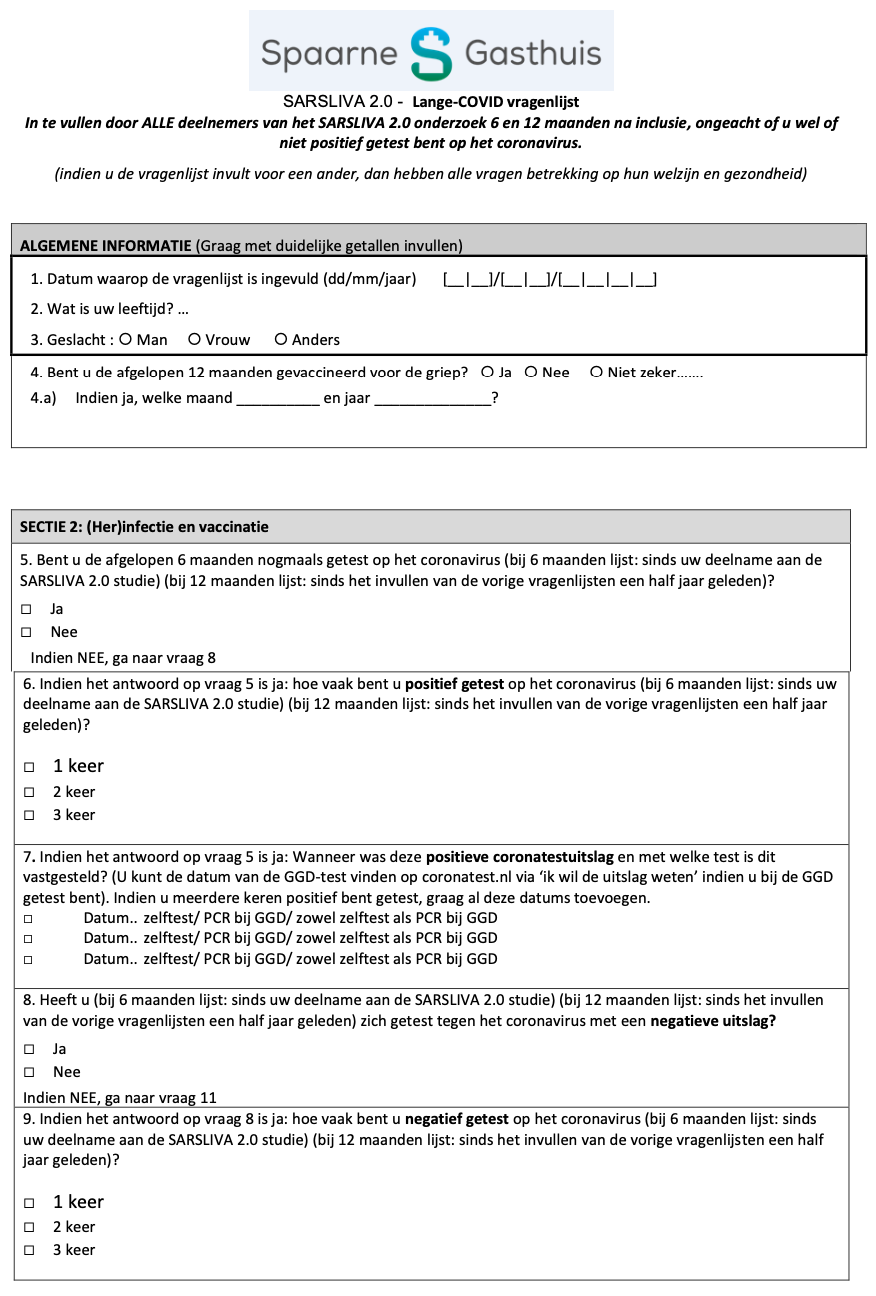


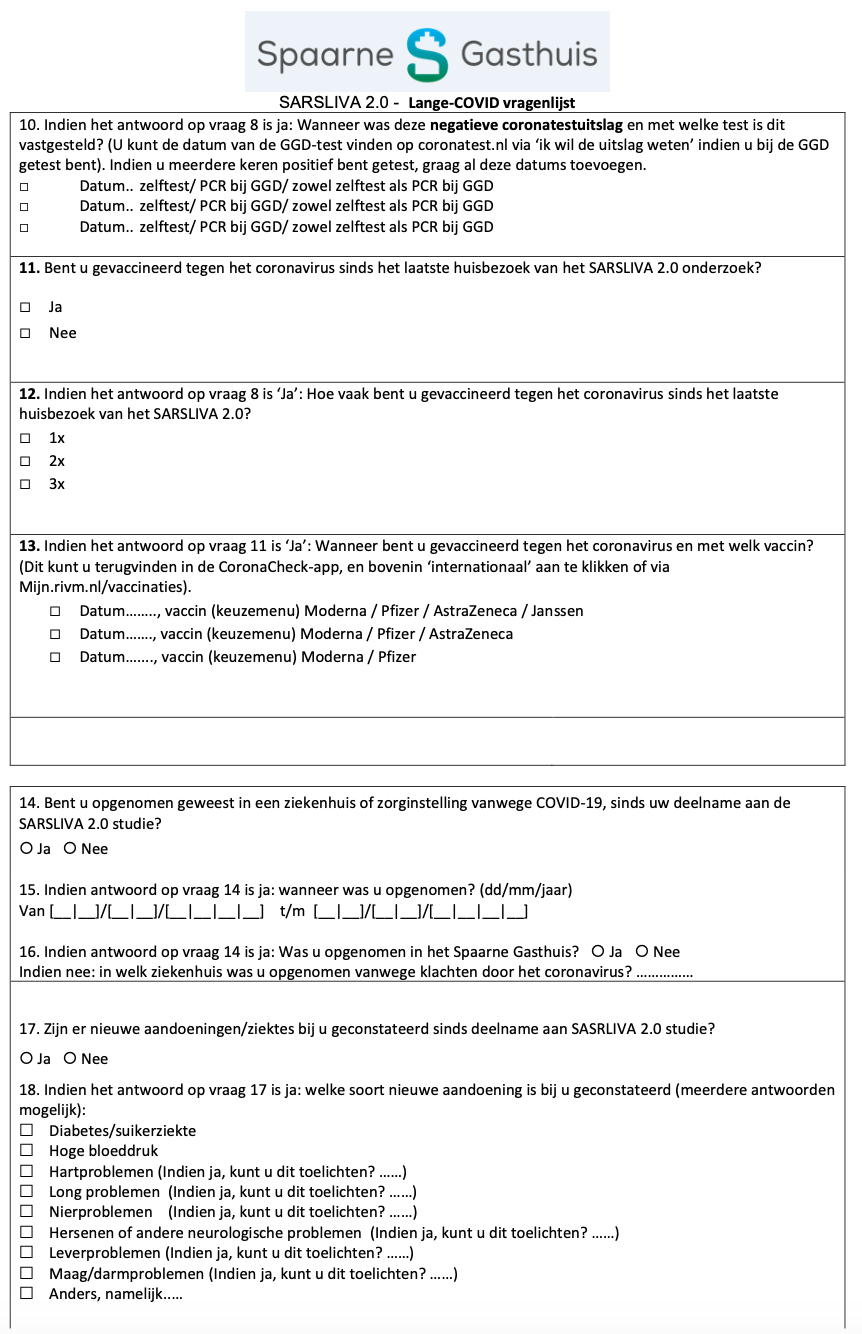


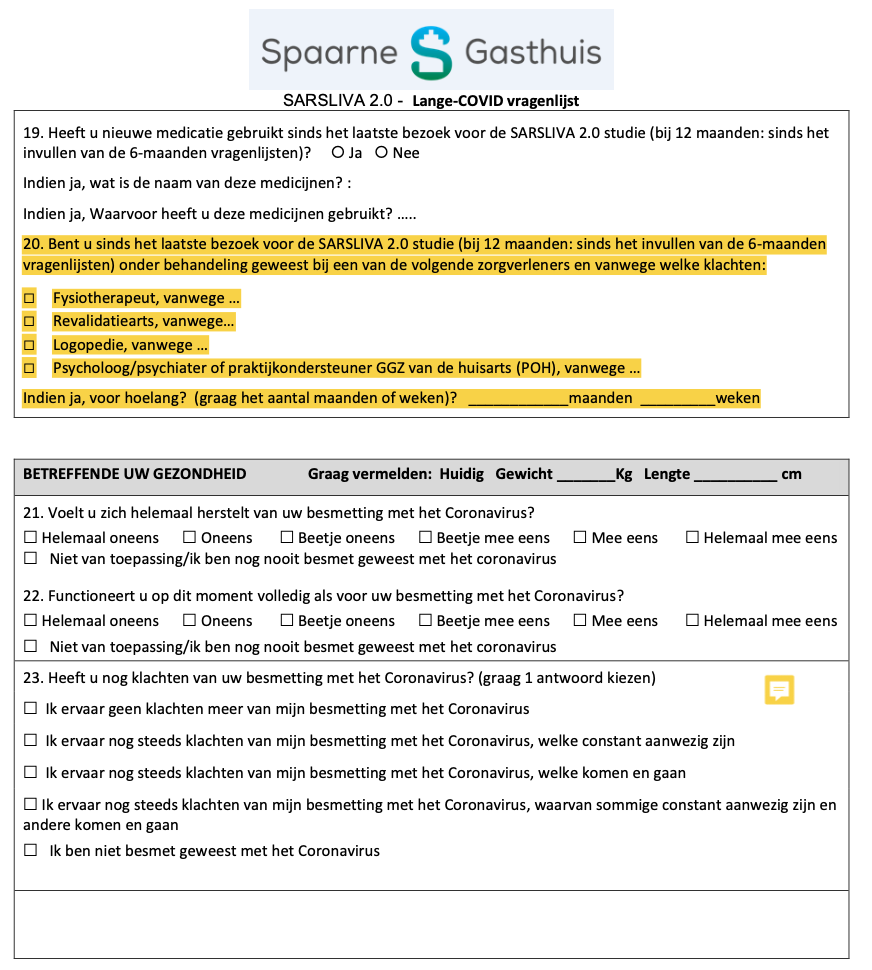


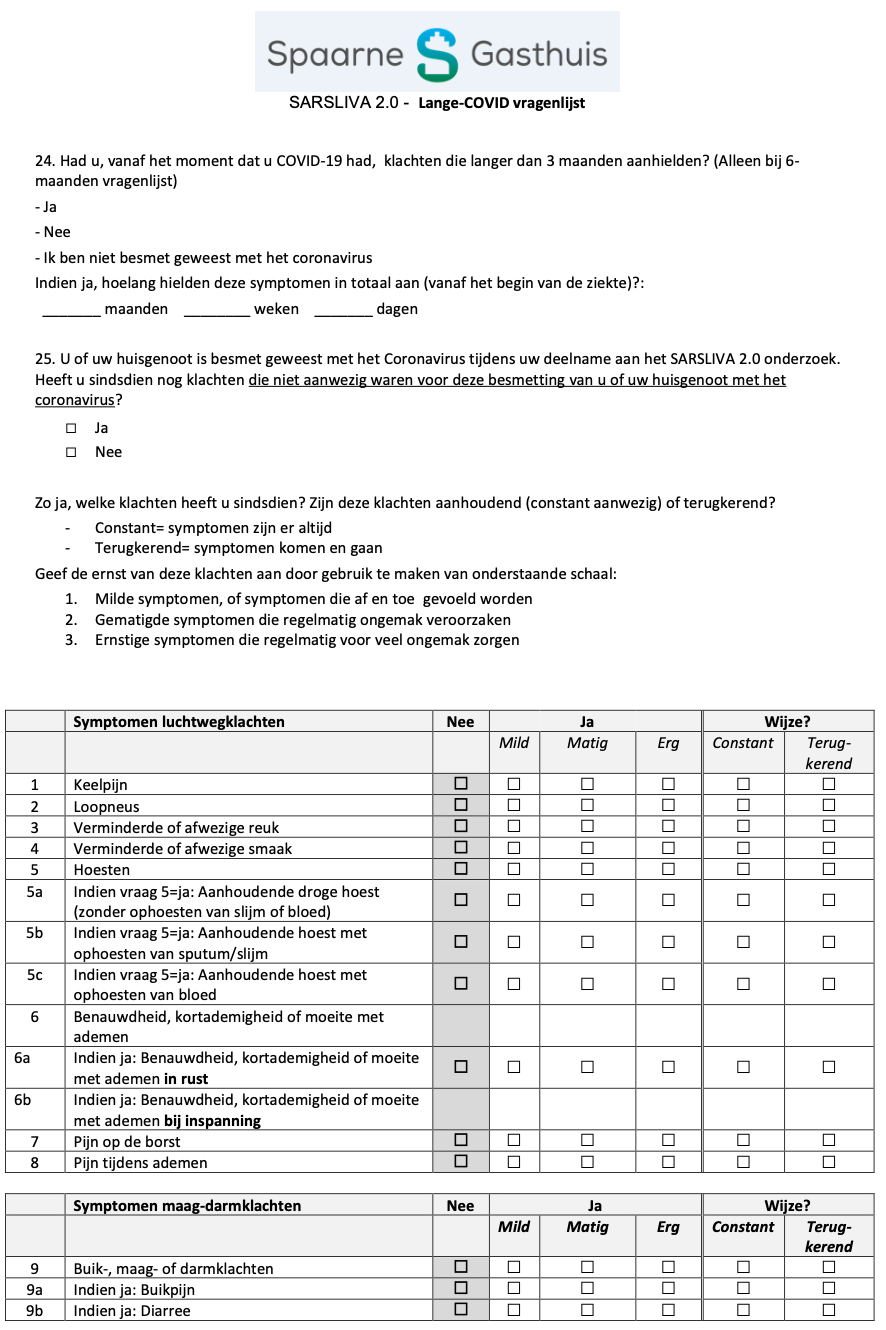


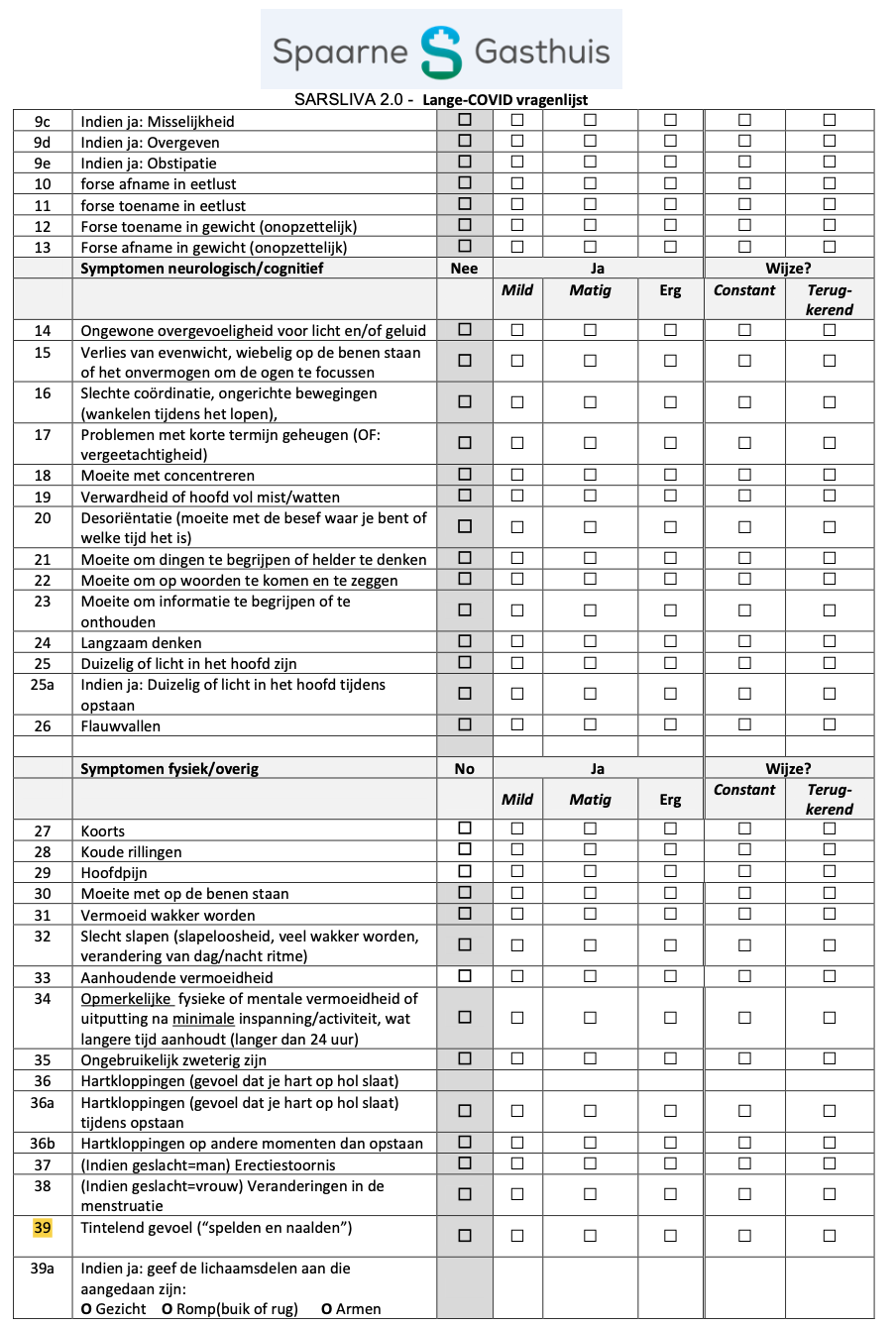


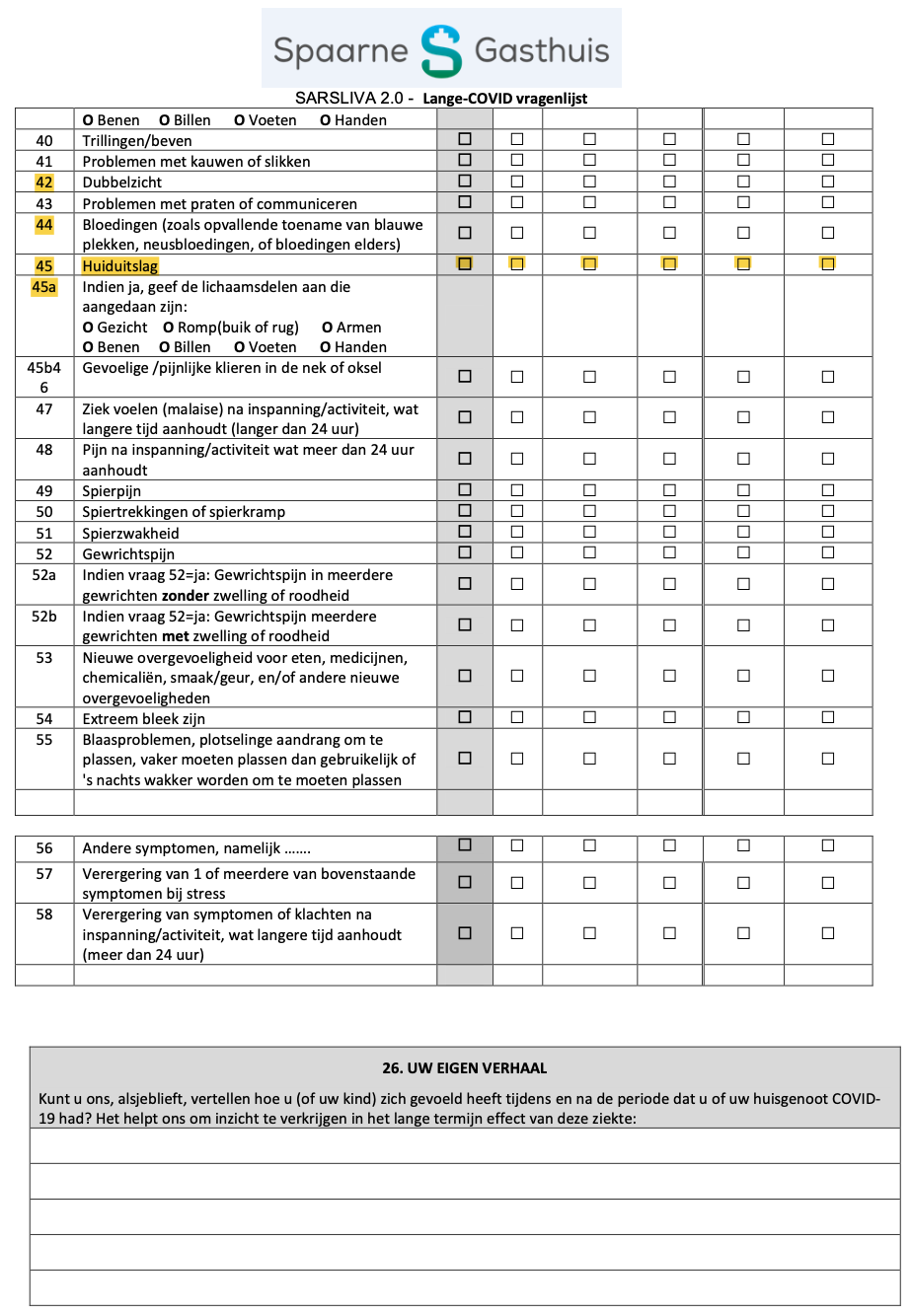


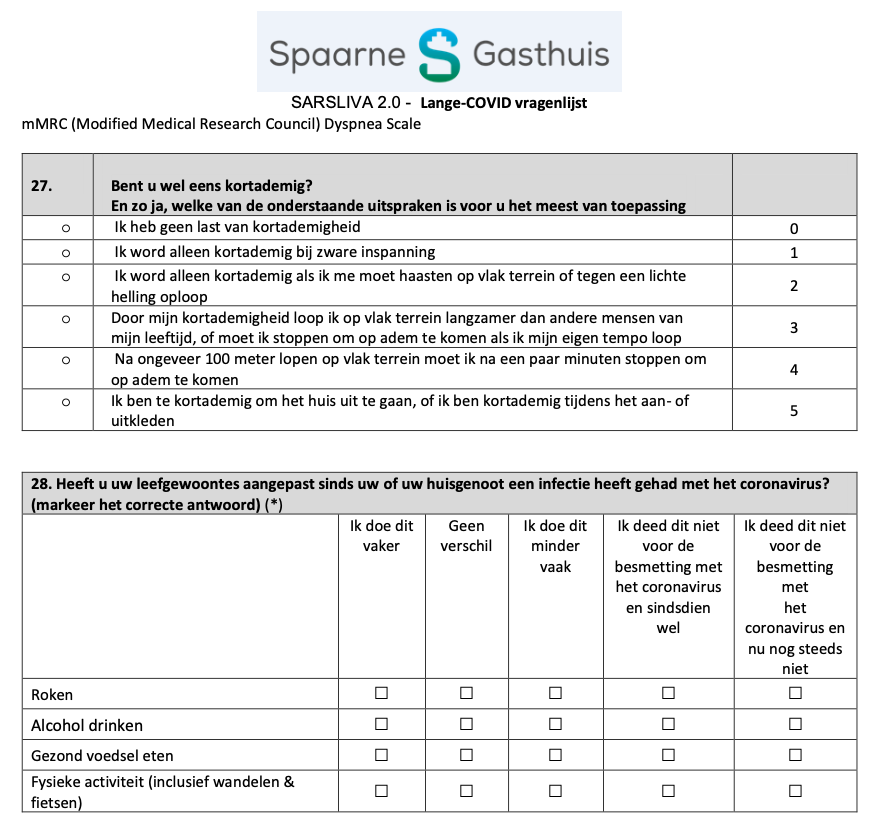


**Supplementary Appendix 2.** Persistent symptoms questionnaire children


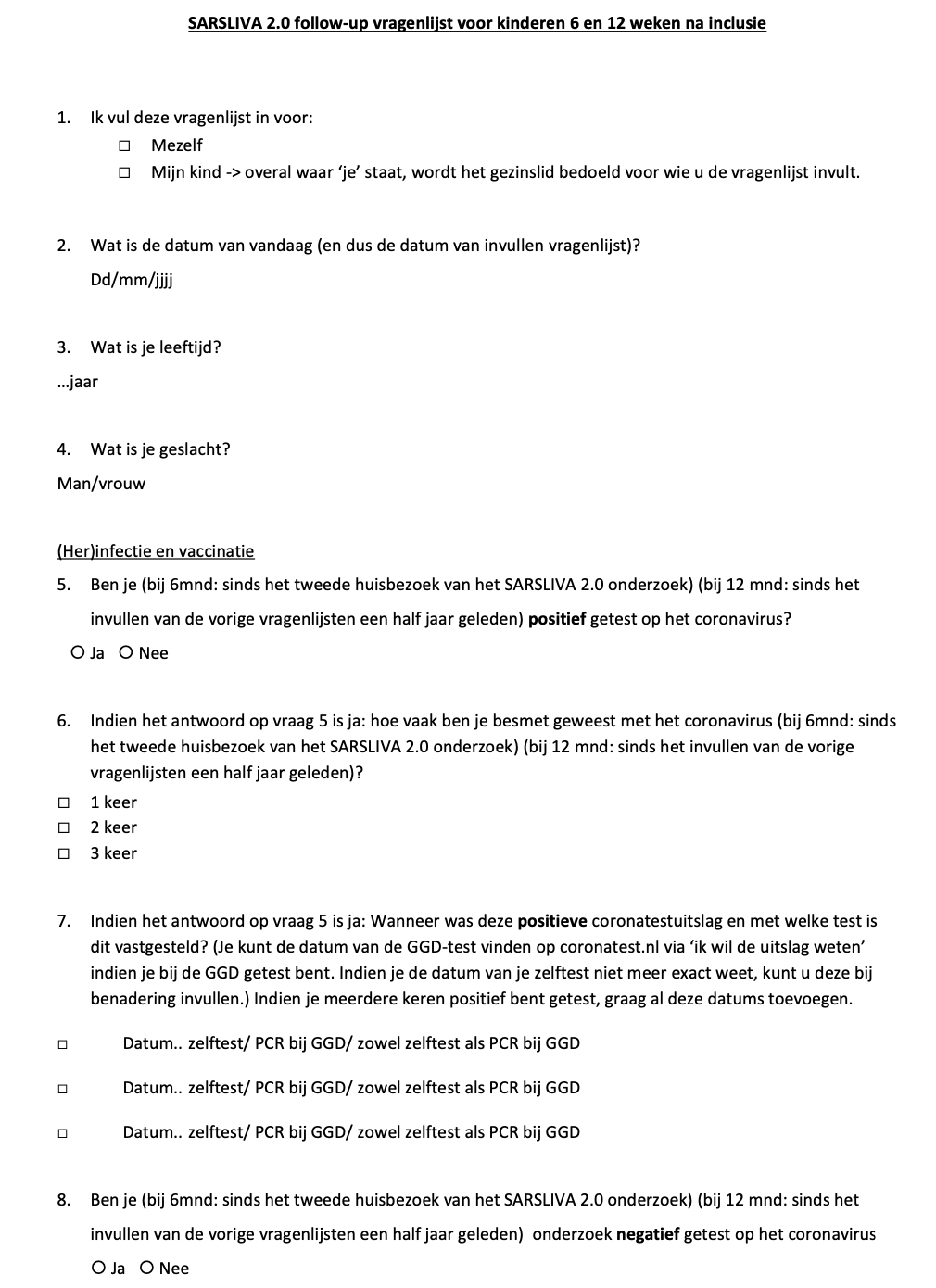


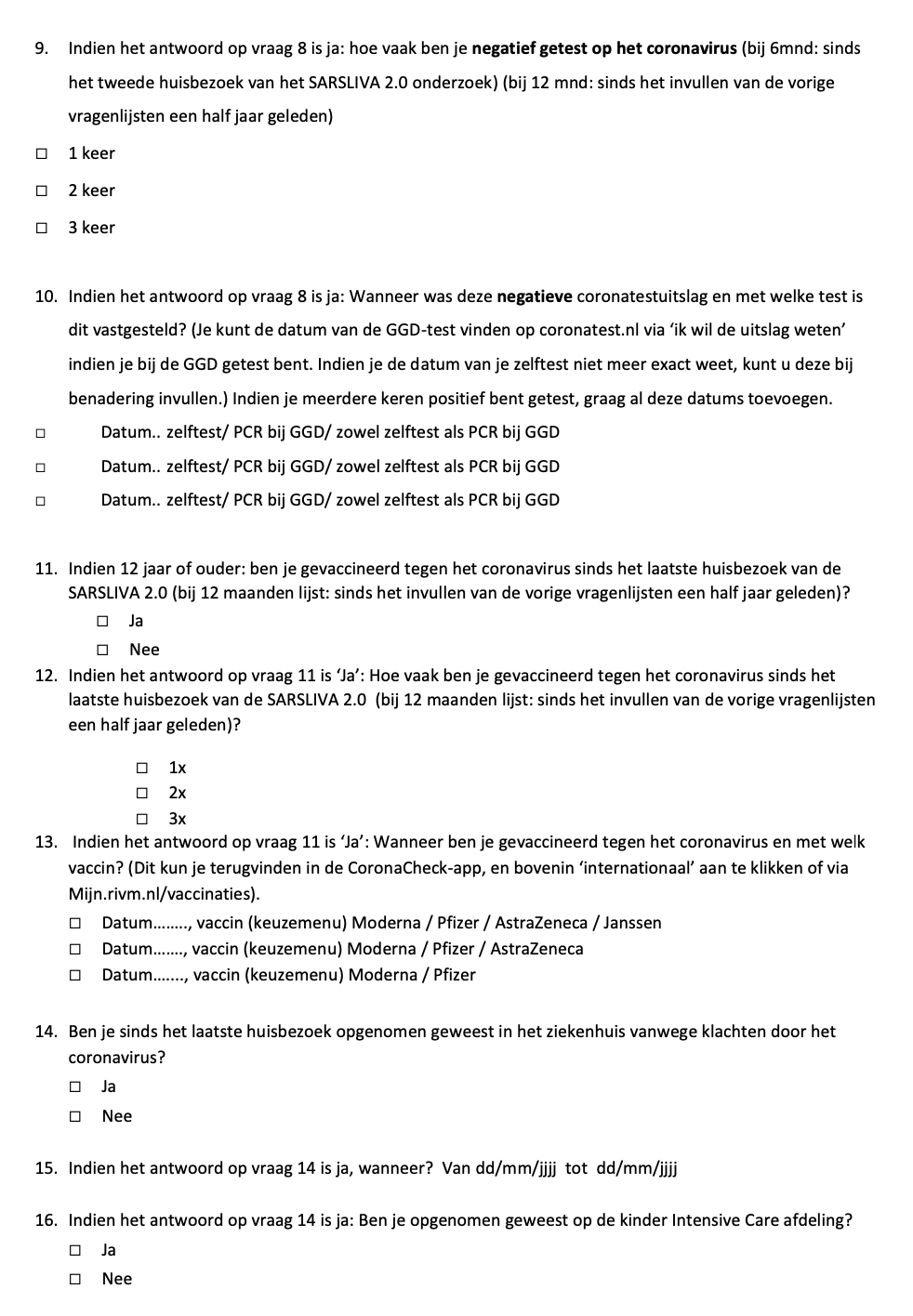

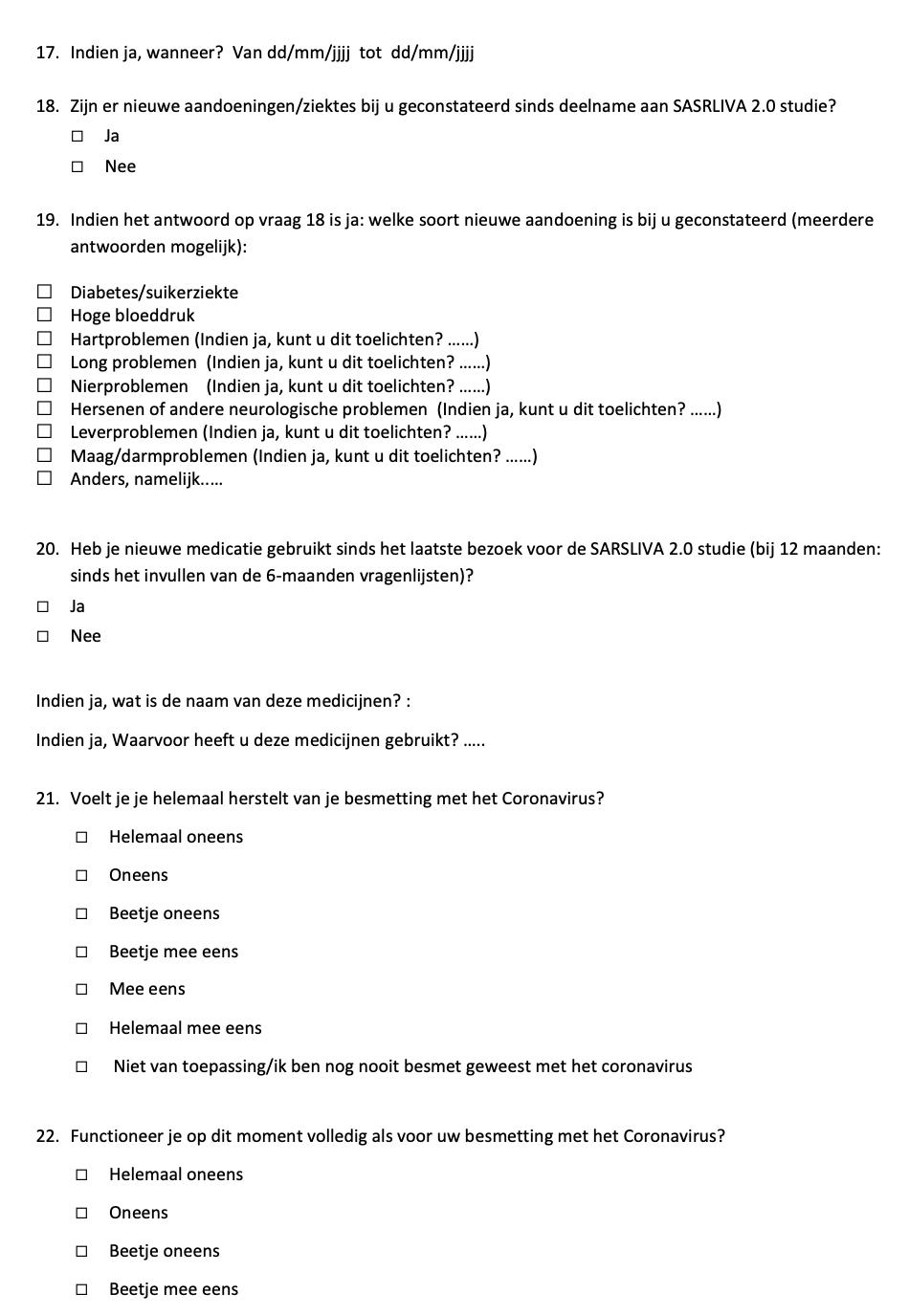


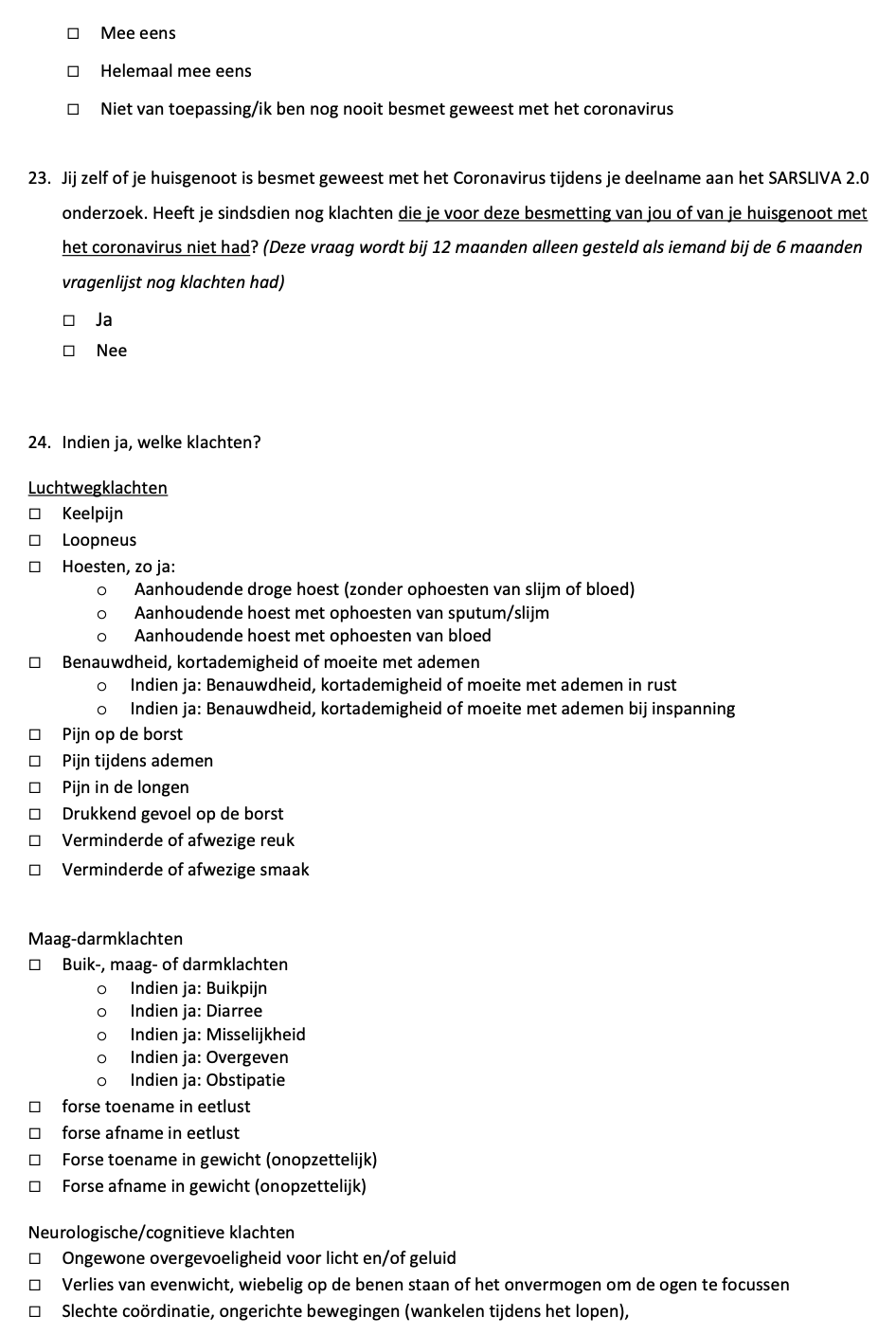


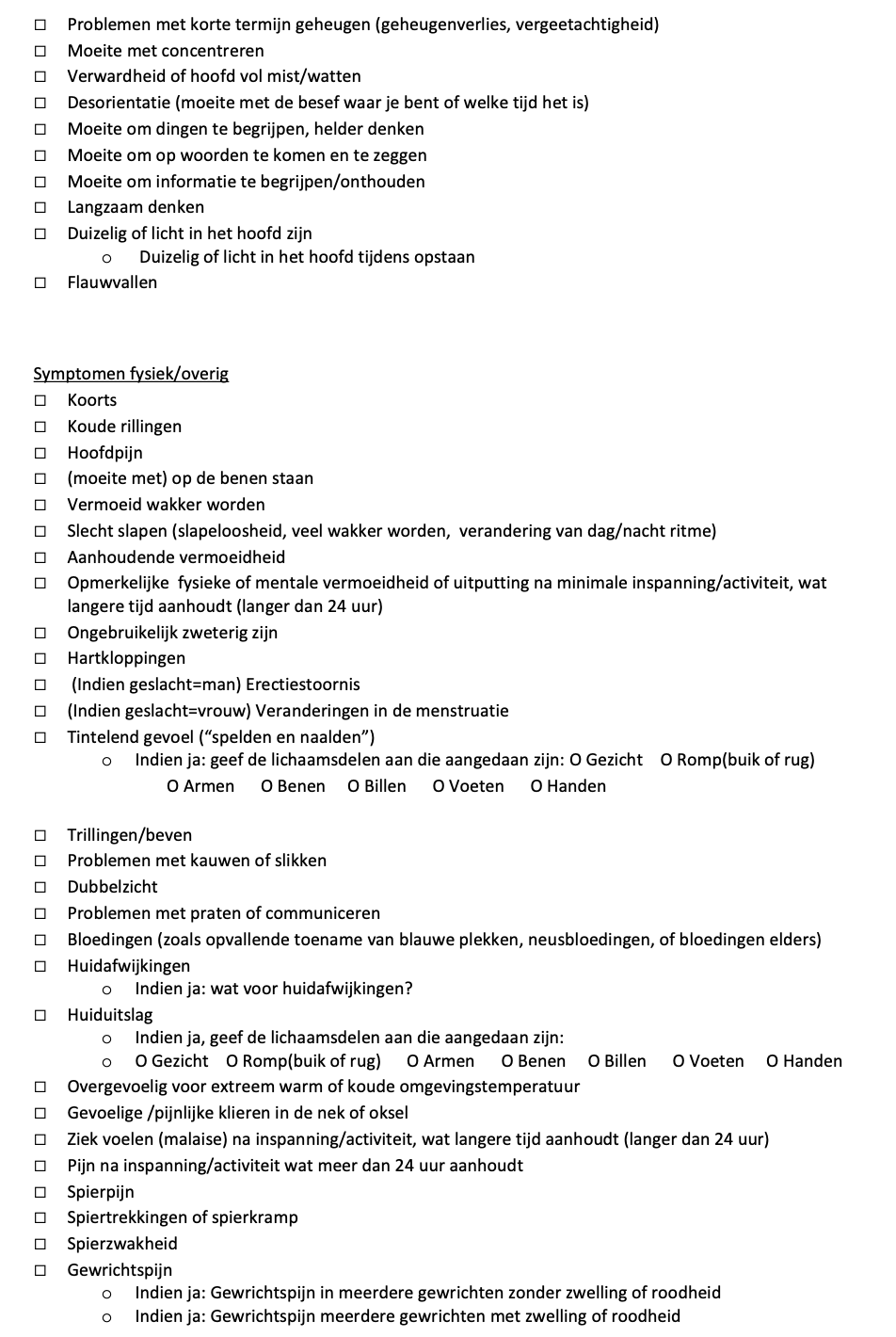

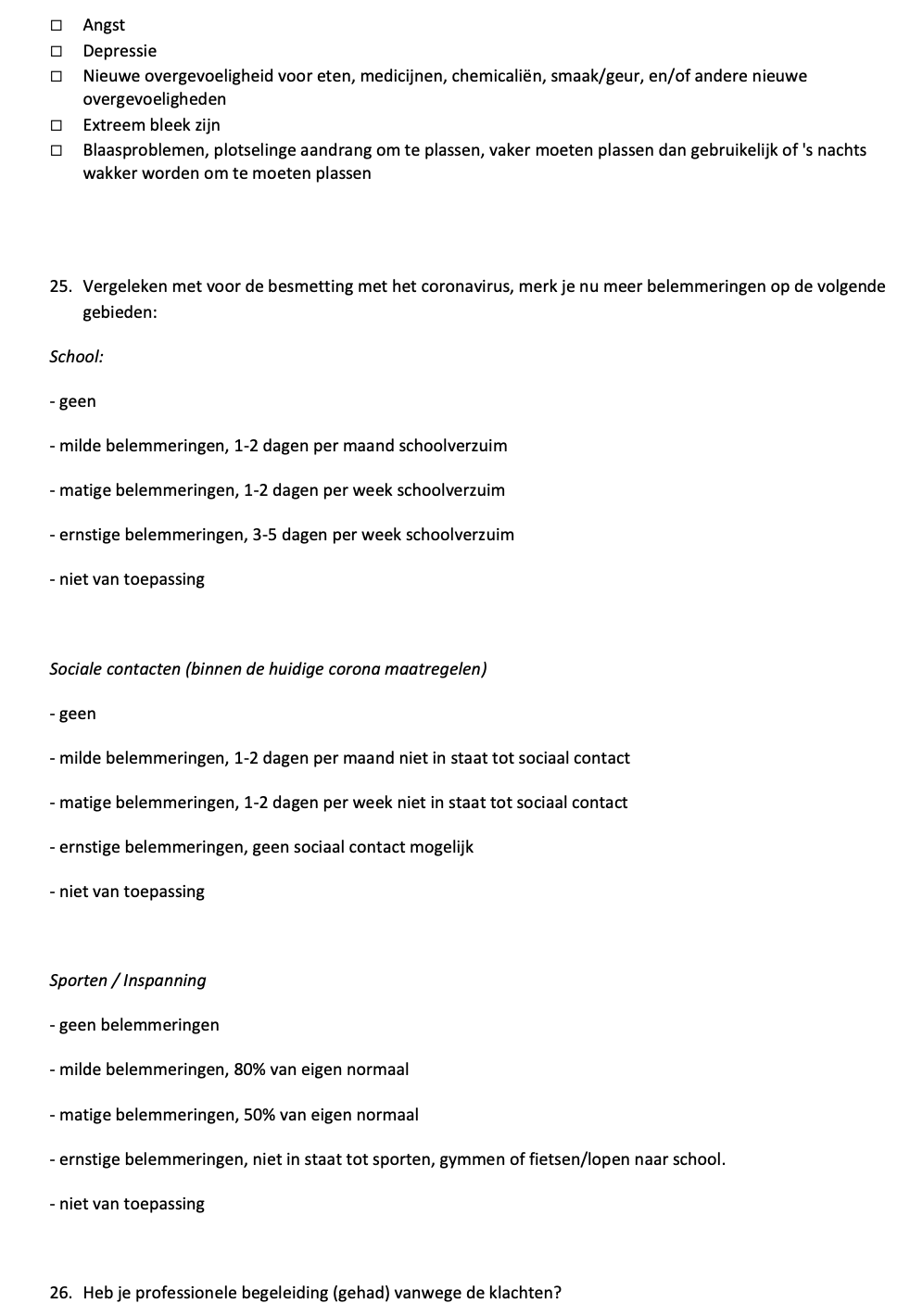


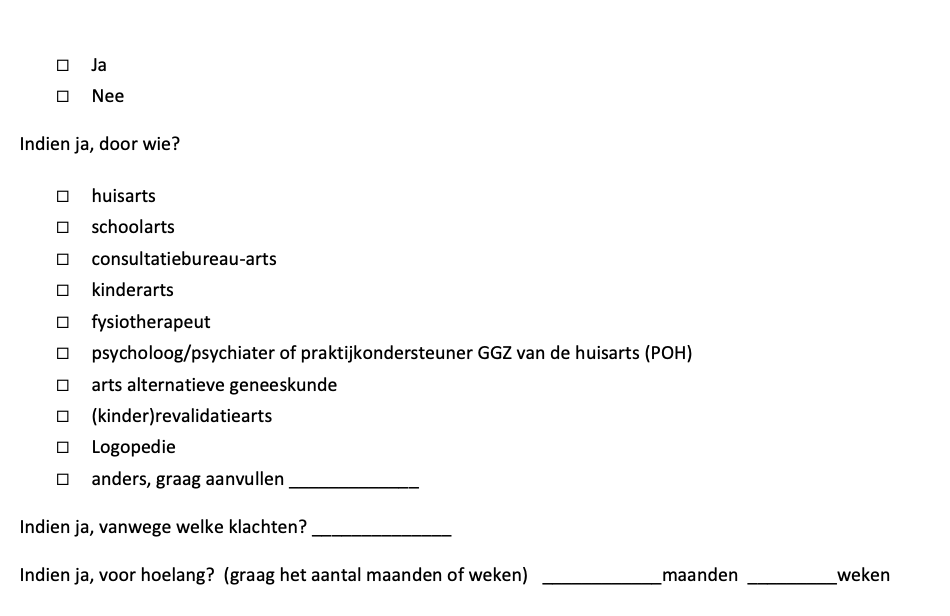

Supplement: Supplementary file 1 — Supplementary_material_Clean. [file JMV-97-e70727-s001.docx]
